# *Xenorhabdus bharatensis* sp. nov.*, Xenorhabdus entomophaga* sp. nov., *Xenorhabdus siamensis* sp. nov., and *Xenorhabdus thailandensis* sp. nov. Isolated from *Steinernema* Entomopathogenic Nematodes

**DOI:** 10.1007/s00284-024-03972-7

**Published:** 2024-11-25

**Authors:** Aunchalee Thanwisai, Ricardo A. R. Machado, Aashaq Hussain Bhat, Sacha J. Pidot, Sarunporn Tandhavanant, Chanakan Subkrasae, Wipanee Meesil, Jiranun Ardpairin, Supawan Pansri, Apichat Vitta

**Affiliations:** 1 Department of Microbiology and Parasitology, Faculty of Medical Science, Naresuan University, Phitsanulok, 65000 Thailand; 2 Centre of Excellence in Medical Biotechnology (CEMB), Faculty of Medical Science, Naresuan University, Phitsanulok, 65000 Thailand; 3 Center of Excellence for Biodiversity, Faculty of Sciences, Naresuan University, Phitsanulok, 65000 Thailand; 4 Experimental Biology Research Group, Institute of Biology, University of Neuchâtel, Rue Emile-Argand 11, 2000 Neuchâtel, Switzerland; 5 Department of Biosciences, University Center for Research and Development, Chandigarh University, Gharuan, 140413 India; 6 Department of Microbiology and Immunology, Doherty Institute, 792 Elizabeth Street, Melbourne, 3000 Australia; 7 Department of Microbiology and Immunology, Faculty of Tropical Medicine, Mahidol University, Bangkok, 10400 Thailand

## Abstract

Four Gram-stain-negative bacterial strains, CS20^T^, AUT15.5^T^, XENO-11^T^, and CCN3.3^T^, isolated from *Steinernema* entomopathogenic nematodes, were found to represent novel species within the genus *Xenorhabdus* (*Gammaproteobacteria, Morganellaceae*). In this study, we described these new species using whole-genome phylogenomic reconstructions, sequence identity values from core genome sequences, and phenotypic characterization. Phylogenetic reconstructions based on 16S rRNA gene sequences showed that: (i) strain CS20^T^ is closely related to *X. stockiae* DSM 17904^T^, (ii) strain AUT15.5^T^ is closely related to *X. budapestensis* DSM 16342^T^, (iii) strain XENO-11^T^ is closely related to *X. khoisanae* DSM 25463^T^, and (iv) strain CCN3.3^T^ is closely related to *X. griffiniae* DSM 17911^T^. The 16S rRNA gene sequence similarity value between strain CS20^T^ and *X. stockiae* DSM 17904^T^ is 97.8%, between strain AUT15.5^T^ and *X. budapestensis* DSM 16342^T^ is 98.1%, between strain XENO-11^T^ and *X. khoisanae* DSM 25463^T^ is 97.8%, and between strain CCN3.3^T^ and *X. griffiniae* DSM 17911^T^ is 98.6%. Phylogenomic reconstructions using whole-genome sequences showed that: (i) strain CS20^T^ is closely related to *X. stockiae* DSM 17904^T^ and *X. innexi* DSM 16336^T^, (ii) strain AUT15.5^T^ is closely related to *X. indica* DSM 17382^T^, (iii) strain XENO-11^T^ is closely related to *X. khoisanae* DSM 25463^T^, and (iv) strain CCN3.3^T^ is closely related to *X. griffiniae* DSM 17911^T^. The digital DNA-DNA hybridization (dDDH) and average nucleotide identity (ANI) values between strains CS20^T^, AUT15.5^T^, XENO-11^T^, and CCN3.3^T^ and the type strains of their more closely related species are below the 70% and the 95–96% divergence thresholds, respectively, used for prokaryotic species delineation. Hence, we propose the following four new species: *Xenorhabdus bharatensis* sp. nov. (the type strain is CS20^T^=CCM 9320^T^=CCOS 2070^T^)*, X. entomophaga* sp. nov. (the type strain is XENO-11^T^=CCM 9389^T^=CCOS 2111^T^), *X. siamensis* sp. nov. (the type strain is AUT15.5^T^=CCM 9405^T^=CCOS 2116^T^), and *X. thailandensis* sp. nov. (the type strain is CCN3.3^T^=CCM 9406^T^=CCOS 2115^T^). The following biochemical tests may be useful for differentiating the novel species from their more closely related taxa: acetoin production, arginine dihydrolase, citrate utilization, gelatinase, glucose oxidation, indole production, and tryptophan deaminase. Our study contributes to a better understanding of the biodiversity and phylogenetic relationships of entomopathogenic bacteria associated with insect parasitic nematodes.

**Supplementary Information:**

The online version contains supplementary material available at 10.1007/s00284-024-03972-7.

## Introduction

Species of the bacterial genus *Xenorhabdus* establish an obligate symbiotic association with *Steinernema* entomopathogenic nematodes (EPNs) [1]. *Steinernema* nematodes are soil-borne organisms that parasitize and reproduce inside insects and certain other small arthropods [1]. These nematodes carry *Xenorhabdus* bacteria inside a specialized vesicle, that is located at the anterior part of the intestine of infective juveniles, and release them immediately after colonizing a host. *Xenorhabdus* bacteria produce a variety of digestive enzymes, toxins, immunosuppressants, and antibiotics that kill and pre-digest the infected host [2]. This environment allows both the nematodes and bacteria to proliferate within the insect cadaver [1]. Once resources are depleted, the nematodes and bacteria re-establish their symbiosis and abandon the cadaver in search of a new host. This symbiotic pair is often used as biological control agents in agricultural settings. In addition, *Xenorhabdus* bacteria serve as model organisms for natural product research [3].

The genus *Xenorhabdus* was proposed by Thomas and Poinar (1979) to accommodate large, Gram-stain-negative, rod-shaped, facultatively anaerobic, entomopathogenic bacteria that form an obligatory symbiosis with *Steinernema* nematodes [4]. Currently, this genus contains 29 taxa with a validly published and correct name [4–20]. In addition, the species names *‘X. anantnagensis’* and *‘X. bakwenae’,* which were recently described, are effectively published and in the validation process [20, 21]. A complete list of species can be found at: https://lpsn.dsmz.de/genus/xenorhabdus [22].

The great application potential of both the nematodes and their symbiotic bacteria in agriculture and biotechnology has motivated several studies aimed at understanding their taxonomy and their biodiversity for several decades already [23]. To this end, collection campaigns are often carried out to isolate novel nematode isolates and to characterize their symbiotic bacteria [8, 9, 23, 24]. In this study in particular, we characterized four *Xenorhabdus* strains isolated from different species of *Steinernema*. As they represent four novel bacterial species, the following names are proposed: *Xenorhabdus bharatensis* sp. nov. (the type strain is CS20^T^=CCM 9320^T^=CCOS 2070^T^)*, X. entomophaga* sp. nov. (the type strain is XENO-11^T^=CCM 9389^T^=CCOS 2111^T^), *X. siamensis* sp. nov. (the type strain is AUT15.5^T^=CCM 9405^T^=CCOS 2116^T^), and *X. thailandensis* sp. nov. (the type strain is CCN3.3^T^=CCM 9406^T^=CCOS 2115^T^). Our study contributes to a better understanding of the biodiversity of this bacterial group, which holds significant agricultural and biotechnological relevance. This advancement enhances our efforts to develop more effective biocontrol tools for sustainable and environmentally friendly agriculture.

## Materials and Methods

### Strain Isolation and Biogeography

The four bacterial strains characterized in this study were isolated from *Steinernema* EPNs. The strain CS20^T^ was isolated from *S. surkhetense* CS20 collected in India [25]. The strain CCN3.3^T^ was isolated from *Steinernema* sp. CCN3.3 collected in Thailand. The strains AUT15.5^T^ and XENO-11^T^ were isolated from two different *Steinernema* isolates, AUT15.5 and XENO-11, collected in Thailand and Switzerland, respectively. These nematodes were lost during the 2020 pandemic and could not be identified to the species level. These nematodes were collected from soil samples using *Galleria mellonella* Stainton (*Lepidoptera: Pyralidae*) larvae as bait. To isolate the bacterial strains, *G. mellonella* larvae were infested with the different nematode strains. Three to four days later, several insect cadavers were surface-sterilized and dissected with a sharp, sterile surgical blade. The internal organs of the insects were spread onto Lysogeny Broth (LB) agar plates (Sigma-Aldrich, Switzerland) and incubated at 28 °C for 48–96 h. *Xenorhabdus*-like colonies were then sub-cultured until monocultures were obtained. Different procedures, such as the characterization of colony and cell morphology, and 16S rRNA gene sequencing (see details below), were carried out to determine culture purity and identity. As we are mainly interested in understanding the taxonomy and biodiversity of *Xenorhabdus,* only strains of this genus were preserved and used for further characterization. The type strains of closely related species were purchased from the Leibniz Institute DSMZ-German Collection of Microorganisms and Cell Cultures using the following strain IDs: *X. griffiniae* DSM 17911^T^, *X. indica* DSM 17382^T^, *X. innexi* DSM 16336^T^, *X. khoisanae* DSM 25463^T^, and *X. stockiae* DSM 17904^T^.

### 16S rRNA Gene Phylogeny and Sequence Comparisons

To determine the taxonomic identities of strains CS20^T^, AUT15.5^T^, XENO-11^T^, and CCN3.3^T^, genomic DNA was first extracted and purified from bacterial monocultures using the GenElute Bacterial Genomic DNA Kit (Sigma–Aldrich, Switzerland) following the manufacturer’s instructions. The resulting DNA was used to amplify 16S rRNA gene sequences via polymerase chain reaction (PCR) with the following universal primers: 27F (5'-AGAGTTTGATCMTGGCTCAG-3') and 1525R (5'-AAGGAGGTGWTCCARCC-3') [26]. The PCR cycling conditions were as follows: an initial denaturation at 94 °C for 10 min, followed by 40 cycles at 94 °C for 30 s, 55 °C for 30 s, and 72 °C for 60 s, with a final extension at 72 °C for 5 min, as described previously [26]. The PCR products were separated by electrophoresis on a 1% TAE-agarose gel stained with GelRed nucleic acid gel stain (Biotium). The bands were then gel-purified (QIAquick Gel Purification Kit, Qiagen), and sequenced by Sanger sequencing (Microsynth AG, Balgach, Switzerland). The resulting 16S rRNA raw sequences were manually curated using Bioedit 7.2.5 [27]. In addition, 16S rRNA gene sequences were obtained directly from whole-genome sequences using the bacterial ribosomal RNA predictor Barrnap 0.7, with the following parameters: reject length threshold=0.5; length cutoff=0.8; and e-value=0.00001 [28]. The sequences obtained were identical to those obtained by Sanger sequencing. The 16S rRNA gene-based phylogenetic relationships were reconstructed using the Neighbor-Joining and the Maximum Likelihood methods based on the Kimura 2-parameter model in MEGA7 [29–31]. Sequences were aligned with MUSCLE (v3.8.31) [32]. The trees are drawn to scale, with branch lengths measured in the number of substitutions per site. Tree supports were determined by the bootstrap method based on 100 replicates. The final data set included a total of 1334 positions. Graphical representation and editing of the phylogenetic trees were performed with Interactive Tree of Life (v3.5.1) [33]. The National Center for Biotechnology Information (NCBI) accession numbers of the sequences used for these analyses are shown in Table S1.

### Genome Sequencing and Genomic Features

The genome sequences of strains CS20^T^ and XENO-11^T^ were obtained as follows. First, genomic DNA was extracted and purified using the GenElute Bacterial Genomic DNA Kit (Sigma–Aldrich, Switzerland) following the manufacturer’s instructions. The purified DNA was then used for library preparation using the TruSeq DNA PCR–Free LT Library Prep Kit (FC–121–3003). Indexed libraries were pooled at equimolar concentrations and sequenced (2×150 bp) on an Illumina HiSeq 3000 instrument. Genomes were assembled using the Bactopia pipeline [34]. Raw Illumina reads were quality trimmed using Trimmomatic 0.39 (options: slidingwindow:4: 8, minlen:127) [35]. The resulting reads were assembled with SPAdes 3.14.1 (-careful, -mismatch-correction, k–mer sizes of 31, 51, 71, 91, and 111 bp) [36]. Scaffolds with a mean read–depth less than 20% of the median read–depth of longer scaffolds (≥ 5000 bp), as well as scaffolds shorter than 200 bp, were removed. Minor assembly errors were corrected using Pilon 1.22 with default parameters [37]. For strains AUT15.5^T^ and CCN3.3^T^, genomic DNA was extracted using the DNeasy Blood & Tissue Kit (Qiagen, Germany). The extracted DNA was prepared for both Nanopore and Illumina sequencing. For long-read sequencing on the MinION platform, DNA was prepared using the Oxford Nanopore Technologies Ligation Sequencing Kit (SQK-LSK109) following the manufacturer's instructions. Prepared libraries were loaded onto a MinION flow cell (Oxford Nanopore Technologies, United Kingdom) and sequenced on a MinION Mk1B device (Oxford Nanopore Technologies, UK) at the Department of Microbiology and Immunology, Doherty Institute, Melbourne, Australia. For short-read sequencing, DNA was prepared using the Nextera XT DNA preparation kit (Illumina) with 150 bp paired ends. The combined MinION and Illumina sequencing data were assembled using the Unicycler hybrid assembler to form a single contig [38]. Completeness and contamination of the assembled genomes were assessed using CheckM v1.1.6 with default parameters [39].

### Genome-Based Phylogenetic Reconstructions and Sequence Comparisons

The Type (Strain) Genome Server (TYGS), a free bioinformatics platform available under https://tygs.dsmz.de, was used to infer whole-genome-based phylogenetic relationships and to calculate whole-genome sequence similarity values [22]. For the phylogenomic inference, all pairwise comparisons among the set of genomes were conducted using Genome BLAST Distance Phylogeny (GBDP) and accurate intergenomic distances inferred under the algorithm ‘trimming’ and distance formula *d*_*5*_, 100 distance replicates were calculated each [40]. The resulting intergenomic distances were used to infer a balanced minimum evolution tree with branch support via FASTME 2.1.6.1 including SPR postprocessing. Branch support was inferred from 100 pseudo-bootstrap replicates each [41]. In addition, core genome-based phylogenetic trees were also constructed. To reconstruct core genome-based phylogenetic relationships, genomes were first aligned using Roary 3.13.0. Genes to be considered core should be presented in 85% of the genomes with an 85% protein identity, and a coverage higher than 90%. The obtained alignments were used to build phylogenomic trees using FastTree 2.1.10 based on the Generalized Time Reversible (GTR) model. Branch support was assessed using the Shimodaira-Hasegawa-like (SH) procedure with 100 replicates. Graphical representation and editing of the phylogenetic trees were performed with Interactive Tree of Life (v3.5.1) [33]. Average nucleotide identify (ANI) values were calculated using FastANI [42]. The NCBI accession numbers of the sequences used for these analyses are shown in Table S1.

### Genomic Comparative Analyses

Genomic comparative analyses were conducted to determine the presence or absence of genes involved in antibiotic resistance and in the production of secondary metabolites. This was achieved by aligning bacterial genomes against the Comprehensive Antibiotic Resistance Database (CARD) [43] and the Antibiotics and Secondary Metabolite Analysis Shell (antiSMASH) database version 7.1.0 [44]. Genes that showed sequence similarity above the threshold of ≥ 70% nucleotide identity were considered present in the genome. Below these thresholds, genes were considered absent or non-functional**.**

### Physiological, Biochemical and Morphological Characterization

To physiologically characterize the *Xenorhabdus* strains, six characteristics were evaluated: dye absorption, Gram staining, optimum temperature for growth, growth on different salt concentrations and pH levels, and antibiotic resistance. Bacterial cultures from single primary colonies were used in all the experiments. The primary forms of the bacteria were determined by assessing their ability to absorb dye from NBTA plates (25 mg/L of bromothymol blue, 4 mg/L of 2,3,5-triphenyltetrazolium chloride, and 20 g/L of nutrient agar). Gram staining was performed with 1 min of Crystal violet, 1 min of Iodine fixing solution, 30 s of 95% alcohol, and 1 min of Safranin O. The optimum temperature for bacterial growth was tested at 20 °C, 24 °C, 28 °C, 30 °C, 37 °C, and 42 °C. Overnight bacterial cultures (100 µL) were spread on LB agar and incubated for 24 h. Growth in the presence of salts was evaluated using three NaCl concentrations: 1% (regular LB medium), 2%, and 3%. Growth at varying pH levels was evaluated at the following pH levels: 3, 5, 7 (regular LB medium), 8, and 9. These tests were performed using 15 mL conical centrifuge tubes, which were inoculated with 5 mL of LB broth and 0.1 mL of an overnight bacterial culture. Subsequently, they were incubated at 28 °C and 180 rpm for 24 h. Antibiotic resistance was evaluated on LB agar supplemented with tetracycline, vancomycin, or gentamicin at a concentration of 30 mg/L. The overnight bacterial culture was measured and adjusted to a cell concentration of 0.5 McFarland using a DEN-1B McFarland densitometer (Biosan, Riga, Latvia) and 0.85% NaCl. Then, the adjusted bacterial suspension (100 µL) was spread on supplemented LB agar and incubated at 28 °C for 24 h. All experiments were performed in triplicate. Biochemical characterizations were conducted in duplicate using the API20E Kit (bioMérieux Inc., Marcy-l'Étoile, France), and the cytochrome oxidase and catalase tests. The API20E strip tests were performed according to the manufacturer’s instructions. Briefly, bacteria were grown on LB agar at 28 °C for 16 h. After that, a single colony was re-suspended in 5 mL of 0.85% NaCl. The bacterial suspension was inoculated into different microtubes containing the dehydrated substrate of each biochemical test. The strips were incubated at 28 °C for 48 h. The oxidase and catalase tests were conducted with 16 h-old liquid LB-bacterial culture. The oxidase test was performed using the filter paper spot method with freshly prepared 1% (v/v) N,N,N',N'-Tetramethyl-p-phenylenediamine·2HCl reagent. The catalase test was executed using the slide method with 3% (v/v) hydrogen peroxide (H_2_O_2_). Lastly, to characterize bacterial morphology, the hanging drop technique was used with cells grown for 16 h at 28 °C on Luria Bertani (LB) broth. Images of bacterial cells were captured using a Leica DM4 B optical microscope equipped with a Leica DFC 7000T Camera (Leica, Wetzlar, Germany). Moreover, the morphology of colonies on LB agar was observed after 48 h incubation.

### Ecological Characterization

To assess the entomopathogenic potential, the different bacterial strains were cultured in LB medium at 28 °C for 16 h. Subsequently, the optical densities of the bacterial cultures were measured at 600 nm using a Shimadzu spectrophotometer (Kyoto, Japan). All cultures were diluted with 0.85% NaCl to achieve an OD_600_ of 1. The bacterial suspensions were subsequently diluted two-fold to obtain four bacterial solutions with OD_600_ values of 1, 0.5, 0.25, and 0.125. Next, 10 μL of each bacterial suspension was injected into 3rd instar *G. mellonella* (waxworm) larvae using a microliter syringe. The waxworms were placed in 9 cm Petri dishes containing filter paper, fed with a small drop of honey and incubated at room temperature. Ten larvae per bacterial strain and dilution were injected (*n* = 10). Mortality was observed every 24 h for 3 days post-injection. Waxworms injected with LB medium and 0.85% NaCl, as well as non-injected waxworms, served as negative controls. Insect survival rates were statistically analysed by repeated measures ANOVA followed by Dunnett’s multiple comparisons tests. All statistical analyses were performed in GraphPad Prism 10.2.3.

### Sequence Data Availability

The whole-genome sequences of *X. bharatensis* sp. nov. CS20^T^, *X. entomophaga* sp. nov. XENO-11^T^, *X. siamensis* sp. nov. AUT15.5^T^, and *X. thailandensis* sp. nov. CCN3.3^T^ were deposited in the National Center for Biotechnology Information (NCBI) databank under the following accession numbers: JBBMXG01, JBBMXH01, JBBMXE01, JBBMXF01, respectively. Additionally, the 16S rRNA gene sequences were deposited under the accession numbers PP544787, PP544789, PP544788, and PP544790, respectively.

## Results and Discussion

The taxonomic positions of four *Xenorhabdus* bacterial strains, CS20^T^, XENO-11^T^, AUT15.5^T^, and CCN3.3^T^, isolated from *Steinernema* nematodes, were determined through molecular and phenotypic analyses. The results indicate that these strains represent four novel species, for which the names *Xenorhabdus bharatensis* sp. nov., *Xenorhabdus entomophaga* sp. nov., *Xenorhabdus siamensis* sp. nov., and *Xenorhabdus thailandensis* sp. nov. are proposed.

### 16S rRNA Gene Phylogeny and Sequence Comparisons

Phylogenetic reconstructions based on 16S rRNA gene sequences showed that: (i) *Xenorhabdus bharatensis* sp. nov. CS20^T^ is closely related to *X. stockiae* DSM 17904^T^, (ii) *X. siamensis* sp. nov. AUT15.5^T^ is closely related to *X. budapestensis* DSM 16342^T^, (iii) *X. entomophaga* sp. nov. XENO-11^T^ is closely related to *X. khoisanae* DSM 25463^T^, and (iv) *X. thailandensis* sp. nov. CCN3.3^T^ is closely related to *X. griffiniae* DSM 17911^T^ (Figs. S1 and S2). The 16S rRNA gene sequence similarity value(s) between *X. bharatensis* sp. nov. CS20^T^ and *X. stockiae* DSM 17904^T^ is 97.8%, between *X. siamensis* sp. nov. AUT15.5^T^ and *X. budapestensis* DSM 16342^T^ is 98.1%, between *X. entomophaga* sp. nov. XENO-11^T^ and *X. khoisanae* DSM 25463^T^ and *X. yunnanensis* XENO-10^T^ are 97.8% and 98.6%, respectively, and between *X. thailandensis* sp. nov. CCN3.3^T^ and *X. griffiniae* DSM 17911^T^ is 98.6% (Fig. S3). Comparatively, lower sequence similarities were observed when the sequences of *X. bharatensis* sp. nov. CS20^T^, *X. entomophaga* sp. nov. XENO-11^T^, *X. siamensis* sp. nov. AUT15.5^T^, and *X. thailandensis* sp. nov. CCN3.3^T^ were compared to the sequences of the type strains of all the species of the genus *Xenorhabdus* with validly published names (Fig. S3)*.*

### Core Genome-Based Phylogenetic Reconstructions and Sequence Comparisons

**Fig. 1:**
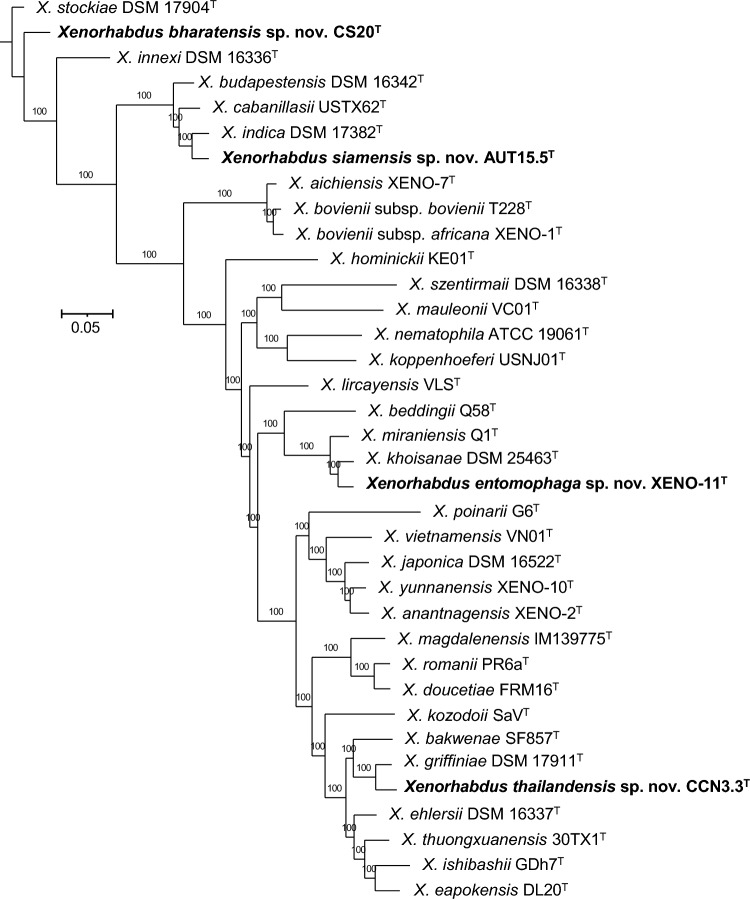
Phylogenomic reconstruction based on core genome sequences of the type strains of all *Xenorhabdus* species with validly published names. A total of 1,430,430 nucleotide positions (1413 core genes) were used in the analyzes. Numbers at the nodes represent SH-like branch supports. Bar represents 0.05 nucleotide substitutions per sequence position. Accession numbers of the genome sequences used for the reconstruction are shown in Table S1

**Fig. 2:**
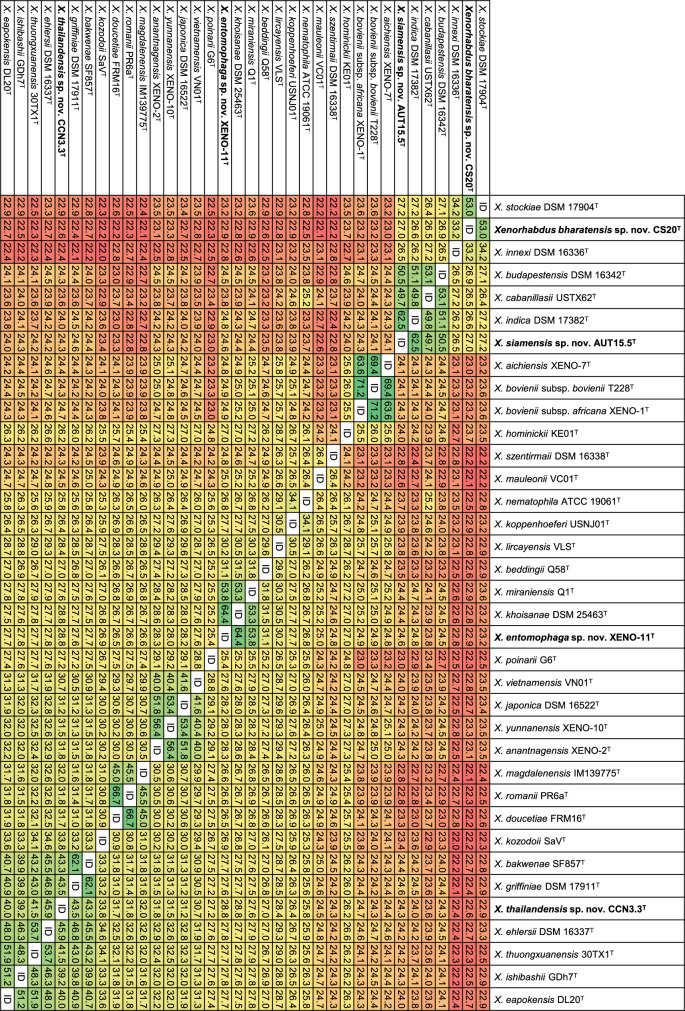
Pairwise comparisons of digital DNA-DNA Hybridization (dDDH) values (%) of the type strains of all *Xenorhabdus* species with validly published names. Accession numbers of gene sequences used are shown in Table S1

Whole-genome-based phylogenomic reconstructions showed that: (i) *X. bharatensis* sp. nov. CS20^T^ is closely related to *X. stockiae* DSM 17904^T^ and to *X. innexi* DSM 16336^T^, (ii) *X. siamensis* sp. nov. AUT15.5^T^ is closely related to *X. indica* DSM 17382^T^, (iii) *X. entomophaga* sp. nov. XENO-11^T^ is closely related to *X. khoisanae* DSM 25463^T^, and (iv) *X. thailandensis* sp. nov. CCN3.3^T^ is closely related to *X. griffiniae* DSM 17911^T^ (Figs. 1, S4). The dDDH value(s) between *X. bharatensis* sp. nov. CS20^T^ and *X. stockiae* DSM 17904^T^ and between *X. bharatensis* sp. nov. CS20^T^ and *X. innexi* DSM 16336^T^ are 53.0% and 33.2%, respectively (Fig. 2). The dDDH value between *X. siamensis* sp. nov. AUT15.5^T^ and *X. indica* DSM 17382^ T^ is 62.5%. The dDDH value between *X. entomophaga* sp. nov. XENO-11^ T^ and *X. khoisanae* DSM 25463^T^ is 64.4%. Lastly, the dDDH value between *X. thailandensis* sp. nov. CCN3.3^T^ and *X. griffiniae* DSM 17911^T^ is 43.5% (Fig. 2). Moreover, the ANI value(s) between *X. bharatensis* sp. nov. CS20^T^ and *X. stockiae* DSM 17904^T^ and between *X. bharatensis* sp. nov. CS20^T^ and *X. innexi* DSM 16336^T^ are 93.1% and 92.1%, respectively (Fig. S5). The ANI value(s) between *X. siamensis* sp. nov. AUT15.5^T^ and *X. indica* DSM 17382^T^ is 90.8%. The ANI value(s) between *X. entomophaga* sp. nov. XENO-11^T^ and *X. khoisanae* DSM 25463^T^ is 86.5%. Lastly, the ANI value(s) between *X. thailandensis* sp. nov. CCN3.3^T^ and *X. griffiniae* DSM 17911^T^ is 92.7% (Fig. S5). Lower sequence similarities were observed when the sequences of *X. bharatensis* sp. nov. CS20^T^*, X. entomophaga* sp. nov. XENO-11^T^, *X. siamensis* sp. nov. AUT15.5^T^, and *X. thailandensis* sp. nov. CCN3.3^T^ were compared to the sequences of the type strains of all the species of the genus *Xenorhabdus* with validly published names. Given that the observed dDDH values are below the 70% threshold and the ANI values are below the 95–96% threshold used for prokaryotic species delineation in general, and for *Xenorhabdus* species delineation in particular, and the clear phylogenetic separations, the strains characterized in this study represent novel species within the genus *Xenorhabdus* [45].

### Genome Sequencing and Genomic Features

The main characteristics of the genomes obtained in this study are summarized in Tables S2, S3, and S4. The main characteristics of the genomes of the novel species are as follows. The genome of *X. bharatensis* sp. nov. CS20^T^ is 4.77 Mb in length, with a 43.4% G+C content and harbors 4228 protein-coding genes. The genome of *X. entomophaga* sp. nov. XENO-11^T^ is 4.96 Mbp in length, with a 43.8% G+C content and harbors 4815 protein-coding genes. The genome of *X. siamensis* sp. nov. AUT15.5^T^ is 4.42 Mbp in length, with a 42.7% G+C content and harbors 3912 protein-coding genes. Lastly, the genome of *X. thailandensis* sp. nov. CCN3.3^T^ is 3.79 Mbp in length, with a 43.2% G+C content and harbors 3508 protein-coding genes (Table S2). The G+C content of all the currently described *Xenorhabdus* species is between 42.7 and 45.7%, the genome length is between 3.18 Mbp and 5.33 Mbp, and the number of protein-coding genes is between 2763 and 4871 (Table S2). The genomes of CS20^T^, XENO-11^T^, AUT15.5^T^, and CCN3.3^T^ are predicted to be 100% complete and contain less than 1.08% of contamination (Table S4).

### Genomic Comparative Analyses

In silico analyses showed the presence of antibiotic resistance genes in the genomes of *X. entomophaga* sp. nov. XENO-11^T^, *X. bharatensis* sp. nov. CS20^T^, *X. siamensis* sp. nov. AUT15.5^T^, and *X. thailandensis* sp. nov. CCN3.3^T^ (Table S5). Furthermore, based on the information provided by antiSMASH, the genomes of novel bacterial species described in this study contain biosynthetic gene clusters responsible for the production of various secondary metabolites. These metabolites include aryl polyenes, bicornutin A1–A2, bovienimide A, carotenoids, fabclavine Ia/Ib/IIa/IIb, gameXpeptide C, holomycin, indigoidine, kleboxymycin, kolossin, luminmide, luminmycin A, putrebactin/avaroferrin, pyrrolizixenamide A, safracin A–B, xenematide, xeneprotides A–C, xenocoumacin I–II, and xenortide A–D (Fig. S6). However, the production of these compounds is expected to vary among species, with some species possessing specific gene clusters for certain metabolites while lacking them for others. For example, only *X. thailandensis* sp. nov. CCN3.3^T^ contains the genes necessary for the production of bicornutin A1–A2, indigoidine, kleboxymycin, safracin A–B, and xenematide. Additionally, only *X. entomophaga* sp. nov. XENO-11^T^ contains the genes required for the production of bovienimide A, gamexpeptide C, holomycin, and kolossin. This suggested a species-specific pattern in the biosynthesis of these secondary metabolites among *Xenorhabdus* bacteria (Fig. S6).

### Physiological and Biochemical Characterization

The following nine species were physiologically and biochemically characterized: *X. bharatensis* sp. nov. CS20^T^, *X. entomophaga* sp. nov. XENO-11^T^, *X. griffiniae* DSM 17911^T^, *X. indica* DSM 17382^T^, *X. innexi* DSM 16336^T^, *X. khoisanae* DSM 25463^T^, *X. siamensis* sp. nov. AUT15.5^T^, *X. stockiae* DSM 17904^T^, and *X. thailandensis* sp. nov. CCN3.3^T^. All these nine bacterial strains are Gram-stain-negative bacilli and absorb dye on NBTA plates. All strains were able to grow at 20 °C, 24 °C, 28 °C, 30 °C, and 37 °C. The growth at 42 °C was highly impaired for all the strains, and totally inhibited for *X. khoisanae* DSM 25463^T^. Additionally, all strains grew in cultures containing 1% NaCl and 2% NaCl. Growth was highly impaired at 3% NaCl. All bacterial strains grew at pH levels of 5, 7, and 8. Growth was highly impaired at a pH of 9, and totally inhibited at a pH of 3. The antibiotic resistance evaluation showed that all strains were susceptible to tetracycline and gentamicin but resistant to vancomycin. Moreover, biochemical tests showed that the four novel species have biochemical capabilities similar to the capabilities of their closely related species (Table 1). All the strains tested were positive or weakly positive for glucose oxidation. All the strains tested were negative for β-galactosidase, catalase, cytochrome oxidase, hydrogen sulfide (H_2_S) production, lysine decarboxylase, NO_2_ production and NO_2_ reduction to N_2_ gas, ornithine decarboxylase, and urease. All the strains tested were negative for the oxidation of amygdalin, arabinose, inositol, mannitol, melibiose, rhamnose, sorbitol, and sucrose. The following biochemical tests may be useful to differentiating the novel species from their more closely related taxa: acetoin production, arginine dihydrolase, citrate utilization, gelatinase, glucose oxidation, indole production, and tryptophan deaminase (Table 1).

**Table 1:** Phenotypic characters that allow to differentiate the type strains of the novel *Xenorhabdus* species described in this study and the type strains of closely related species

	1	2	3	4	5	6	7	8	9
Acetoin production	+	−	−	+	+	+	−	+	+
Arginine dihydrolase	w+	−	w+	w+	w+	w+	−	−	−
Citrate utilization	−	+	−	−	−	−	−	−	+
Gelatinase	+	w+	+	+	+	+	−	+	+
Glucose oxidation	w+	+	+	w+	w+	+	w+	+	+
Indole production	−	−	−	−	+	−	−	−	−
Tryptophan deaminase	−	−	−	−	−	−	−	+	−

1: *X. bharatensis* sp. nov. CS20^T^; 2: *X. entomophaga* sp. nov. XENO-11^T^; 3: *X. griffiniae* DSM 17911^T^; 4: *X. indica* DSM 17382^T^; 5: *X. innexi* DSM 16336^T^; 6: *X. khoisanae* DSM 25463^T^; 7: *X. siamensis* sp. nov. AUT15.5^T^; 8: *X. stockiae* DSM 17904^T^; 9: *X. thailandensis* sp. nov. CCN3.3^T^.+: positive reaction, − : negative reaction, w+: weakly positive reaction

### Ecological Characterization

When bacterial suspensions of varying concentrations were injected into the hemocoel of waxworms, all *Xenorhabdus* strains showed a high potential to kill the larvae (Fig. S7–S9). Even at the lowest concentration (OD_600_ = 0.125), the larval mortality rate exceeded 80% within 24 h post-injection. Notably, *Xenorhabdus thailandensis* sp. nov. CCN3.3^T^, *X. bharatensis* sp. nov. CS20^T^, *X. entomophaga* sp. nov. XENO-11^T^, *X. indica* DSM 17382^T^, *X. khoisanae* DSM 25463^T^, and *X. griffiniae* DSM 17911^T^ were the most pathogenic strains, as they killed 100% of the insects at all concentrations tested within 24 h post-injection (Fig. S7–S9).

### Taxonomic Conclusions

Based on the results of this polyphasic taxonomic study, we propose the establishment of the following novel species: *Xenorhabdus bharatensis* sp. nov. (the type strain is CS20^T^ = CCM 9320^T^ = CCOS 2070^T^)*, X. entomophaga* sp. nov. (the type strain is XENO-11^T^ = CCM 9389^T^ = CCOS 2111^T^), *X. siamensis* sp. nov. (the type strain is AUT15.5^T^ = CCM 9405^T^ = CCOS 2116^T^), and *X. thailandensis* sp. nov. (the type strain is CCN3.3^T^ = CCM 9406^T^ = CCOS 2115^T^).

## Protologues

### Description of *Xenorhabdus bharatensis* sp. nov.

(bha.rat.en’sis. N.L. fem. adj. *bharatensis* derived from Bhārat the hindi name for India, the country where the nematode hosting the type strain was isolated). Cells are rod-shaped. Approximate cell size is 1.2×7.8 µm. On LB agar plates, colonies appear cream or light yellow and range in size from 0.3–0.8 cm in diameter after 48 h of growth. Growth is observed at temperatures between 20 and 42 °C. Optimal growth occurs at 28–30 °C. Bacterial growth occurs at pH between 5 and 9 (optimum 5–7) and not at pH 3. Bacterial growth occurs in LB medium containing 1–3% NaCl (optimum 1–2%). Positive for acetoin production, and gelatinase. Weak positive for arginine dihydrolase, and glucose oxidation. Negative for β-galactosidase, catalase, citrate utilization, cytochrome oxidase, lysine decarboxylase, ornithine decarboxylase, tryptophan deaminase, and urease. Does not produce hydrogen sulfide, indole, or nitrogen dioxide, and does not reduce nitrogen dioxide to nitrogen gas. Does not oxidize arabinose, amygdalin, inositol, mannitol, melibiose, rhamnose, sorbitol, or sucrose. The type strain CS20^T^ (=CCM 9320^T^=CCOS 2070^T^) was isolated from *Steinernema surkhetense* CS20 isolated from soil samples collected in the Bijnor district of Western part of Uttar Pradesh (India). The type strain genome length is 4.77 Mbp, with 43.4% G+C content, and the genome harbors 4228 protein-coding genes.

### Description of *Xenorhabdus entomophaga* sp. nov.

(en.to.mo.pha’ga. Gr. neut. adj. *entomon*, segmented, used to refer to insects; from Gr. fem. adj. suff. -*phagos*, eater; N.L. fem. adj. *entomophaga*, insect eater). Cells are rod-shaped, approximately 1.0×3.8 µm in size. Colonies are cream or light yellow on LB agar plates, measuring about 0.3–0.7 cm in diameter after 5–7 days of growth on LB agar plates. Growth is observed at temperatures between 20 and 42 °C. Optimal growth occurs at 28–30 °C. Bacterial growth occurs at pH between 5 and 9 (optimum 5–7) and not at pH 3. Bacterial growth occurs in LB medium containing 1–3% NaCl (optimum 1–2%). Positive for citrate utilization. Weak positive for gelatinase. Oxidizes glucose but does not oxidize amygdalin, arabinose, inositol, melibiose, rhamnose, sorbitol, or sucrose. Negative for acetoin production, arginine dihydrolase, β-galactosidase, catalase, cytochrome oxidase, lysine decarboxylase, ornithine decarboxylase, tryptophan deaminase, and urease. Does not produce hydrogen sulfide, indole, or nitrogen dioxide. Does not reduce nitrogen dioxide to nitrogen gas. The type strain XENO-11^T^ (= CCM 9389^T^ = CCOS 2111^T^) was isolated from a *Steinernema* nematode. The type strain genome is 4.96 Mbp in length, with 43.8% G+C content, and the genome harbors 4815 protein-coding genes.

### Description of *Xenorhabdus siamensis* sp. nov.

(si.am.en’sis. *Siam*, old name of Thailand; L. masc./fem. adj. suff. -*ensis*, indicating geographical origin; N.L. masc./fem. adj. *siamensis*, of or pertaining to Siam (Thailand), where the type strain was isolated). Cells are rod-shaped. Approximate cell size is 1.3×8.6 µm. Colonies are light yellowish brown in colour on LB agar plates. Colonies are of about 0.1–0.5 cm in diameter after 5–7 days growth on LB agar plates. Growth is observed at temperatures between 20 and 42 °C. Optimal growth occurs at 28–30 °C. Bacterial growth occurs at pH between 5 and 9 (optimum 5–7) and not at pH 3. Bacterial growth occurs in LB medium containing 1–3% NaCl (optimum 1–2%). Weak positive for glucose oxidization. Negative for acetoin production, arginine dihydrolase, β-galactosidase, catalase, citrate utilization, cytochrome oxidase, gelatinase, lysine decarboxylase, ornithine decarboxylase, tryptophan deaminase, and urease. Does not oxidize amidgdalin, arabinose, inositol, mannitol, melibiose, rhamnose, sorbitol, or sucrose. Does not produce hydrogen sulfide, indole, or nitrogen dioxide. Does not reduce nitrogen dioxide to nitrogen. The type strain AUT15.5^T^ (= CCM 9405^T^ = CCOS 2116^T^) was isolated from a *Steinernema* nematode isolated from soil samples collected in Muang District, Uttaradit Province of lower northern part of Thailand. The type strain genome length is 4.42 Mbp, with 42.7% G+C content, and the genome harbors 3912 protein-coding genes.

### Description of *Xenorhabdus thailandensis* sp. nov.

(thai.lan.den’sis. N.L. masc./fem. adj. *thailandensis*, pertaining to Thailand, where the type strain was isolated). Cells are rod-shaped. Approximate cell size is 1.5×8.5 µm. Colonies are light yellowish brown in colour on LB agar plates. Colonies are of about 0.2–0.4 cm in diameter after 5–7 days growth on LB agar plates. Growth is observed at temperatures between 20 and 42 °C. Optimal growth occurs at 28–30 °C. Bacterial growth occurs at pH between 5 and 9 (optimum 5–7) and not at pH 3. Bacterial growth occurs in LB medium containing 1–3% NaCl (optimum 1–2%). Positive for acetoin production, citrate utilization, and gelatinase. Oxidizes glucose but does not oxidize amidgdalin, arabinose, inositol, mannitol, melibiose, rhamnose, sorbitol, or sucrose. Negative for arginine dihydrolase, β-galactosidase, catalase, cytochrome oxidase, lysine decarboxylase, ornithine decarboxylase, tryptophan deaminase, and urease. Does not produce hydrogen sulfide, indole, or nitrogen dioxide. Does not reduce nitrogen dioxide to nitrogen gas. The type strain CCN3.3^T^ (= CCM 9406^T^ = CCOS 2115^T^) was isolated from a *Steinernema* sp. CCN3.3 nematode isolated from soil samples collected in Manorom District, Chai Nat Province of Central part of Thailand. The type strain genome is 3.79 Mbp in length, with 43.2% G+C content, and harbors 3508 protein-coding genes.

## Supplementary Information

Below is the link to the electronic supplementary material.

Supplementary file1 (PDF 1386 KB)
