# Supplementary material for: Xenorhabdus bharatensis sp. nov., Xenorhabdus entomophaga sp. nov., Xenorhabdus siamensis sp. nov., and Xenorhabdus thailandensis sp. nov. Isolated from Steinernema Entomopathogenic Nematodes
Source: Curr Microbiol. 2024 Nov 25;82(1):10. doi: 10.1007/s00284-024-03972-7 (PMC11588968; doi:10.1007/s00284-024-03972-7)
Supplement: Supplementary file 1 — Supplementary file1 (PDF 1386 KB) [file 284_2024_3972_MOESM1_ESM.pdf]

-SUPPLEMENTARY MATERIAL-

Xenorhabdus bharatensis sp. nov., *Xenorhabdus entomophaga* sp. nov., *Xenorhabdus siamensis* sp. nov., and *Xenorhabdus thailandensis* sp. nov. isolated from *Steinernema* entomopathogenic nematodes

Aunchalee Thanwisai[1,2,3+*], Ricardo A. R. Machado[4+*], Aashaq Hussain Bhat[4,5] , Sacha J. Pidot[6], Sarunporn Tandhavanant[7], Chanakan Subkrasae[1],Wipanee Meesil[1], Jiranun Ardpairin[1], Supawan Pansri[1], Apichat Vitta[1,2,3]

[1]*Department of Microbiology and Parasitology, Faculty of Medical Science, Naresuan University, Phitsanulok 65000, Thailand.*

[2]*Centre of Excellence in Medical Biotechnology (CEMB), Faculty of Medical Science, Naresuan University, Phitsanulok, 65000, Thailand.*

[3]*Center of Excellence for Biodiversity, Faculty of Sciences, Naresuan University, Phitsanulok, 65000, Thailand.*

[4]*Experimental Biology Research Group, Institute of Biology, University of Neuchâtel, Neuchâtel 2000, Switzerland.*

[5]*Department of Biosciences, University Center for Research and Development, Chandigarh University, Gharuan 140413, India.*

[6]*Department of Microbiology and Immunology, Doherty Institute, 792 Elizabeth Street, Melbourne, 3000 Australia*

[7]*Faculty of Tropical Medicine, Department of Microbiology and Immunology, Mahidol University, Bangkok, 10400, Thailand.*

+: equal contribution

[*]: Corresponding authors:

Ricardo A. R. Machado (rarm.machado@gmail.com). Experimental Biology Research Group. Institute of Biology. University of Neuchâtel. Rue Emile-Argand 11, 2000 Neuchâtel, Switzerland. ORCID: 0000-0002-7624-1105.

Aunchalee Thanwisai (aunchaleet@nu.ac.th). Department of Microbiology and Parasitology, Faculty of Medical Science, Naresuan University, Phitsanulok 65000, Thailand.

-SUPPLEMENTARY FIGURES-

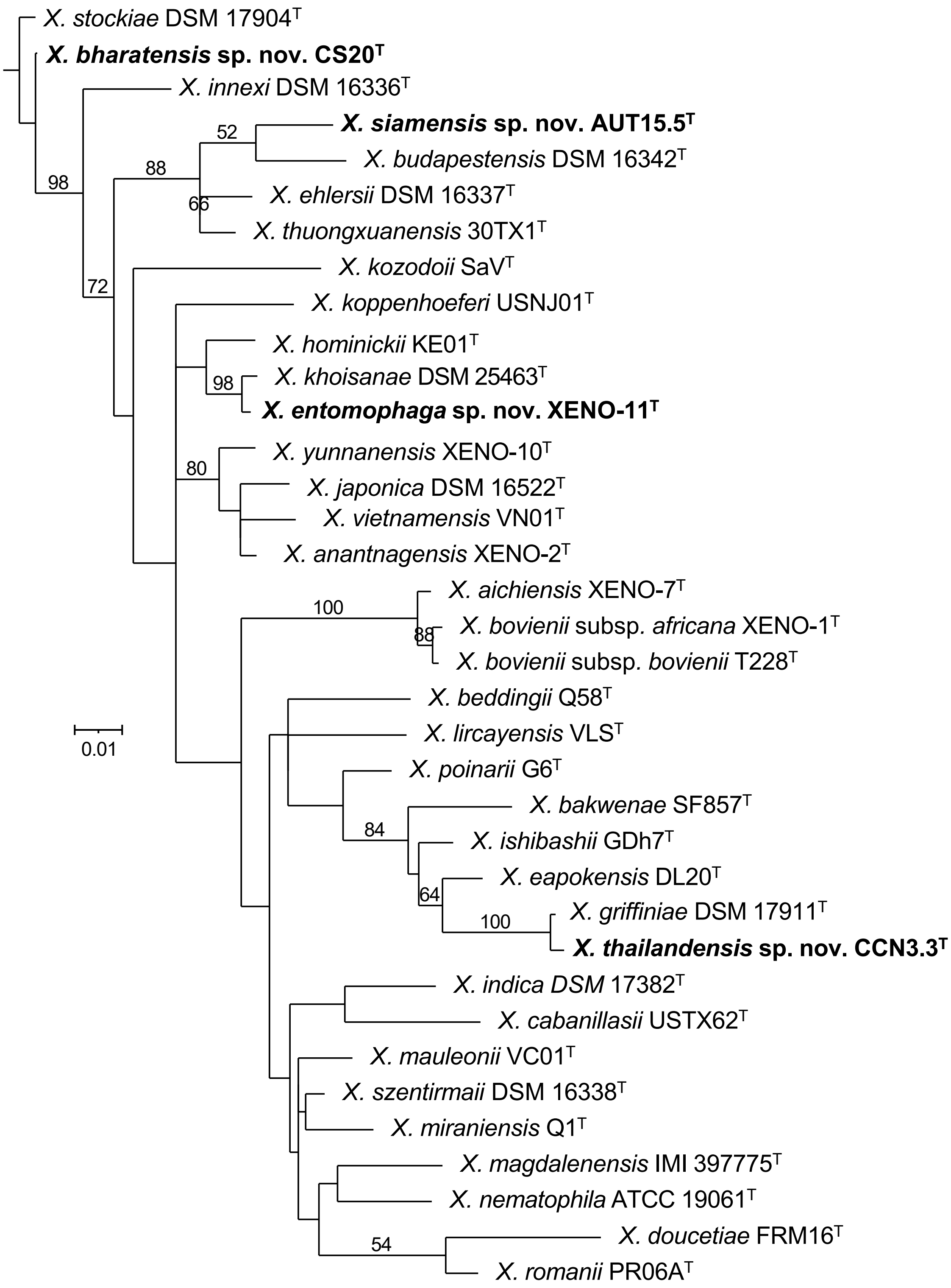

Figure S1. Maximum-likelihood phylogenetic tree reconstructed from the 16S rRNA gene sequences of the type strains of all *Xenorhabdus* species with validly published names. The evolutionary history was inferred by using the Maximum Likelihood method based on the Kimura 2-parameter model. The tree with the highest log likelihood is shown. The percentage of trees in which the associated taxa clustered together is shown next to the branches. The tree is drawn to scale, with branch lengths measured in the number of substitutions per site. There were a total of 1334 positions in the final dataset. Evolutionary analyses were conducted in MEGA7 based on 100 replications. Accession numbers of used gene sequences are shown in Table S1.

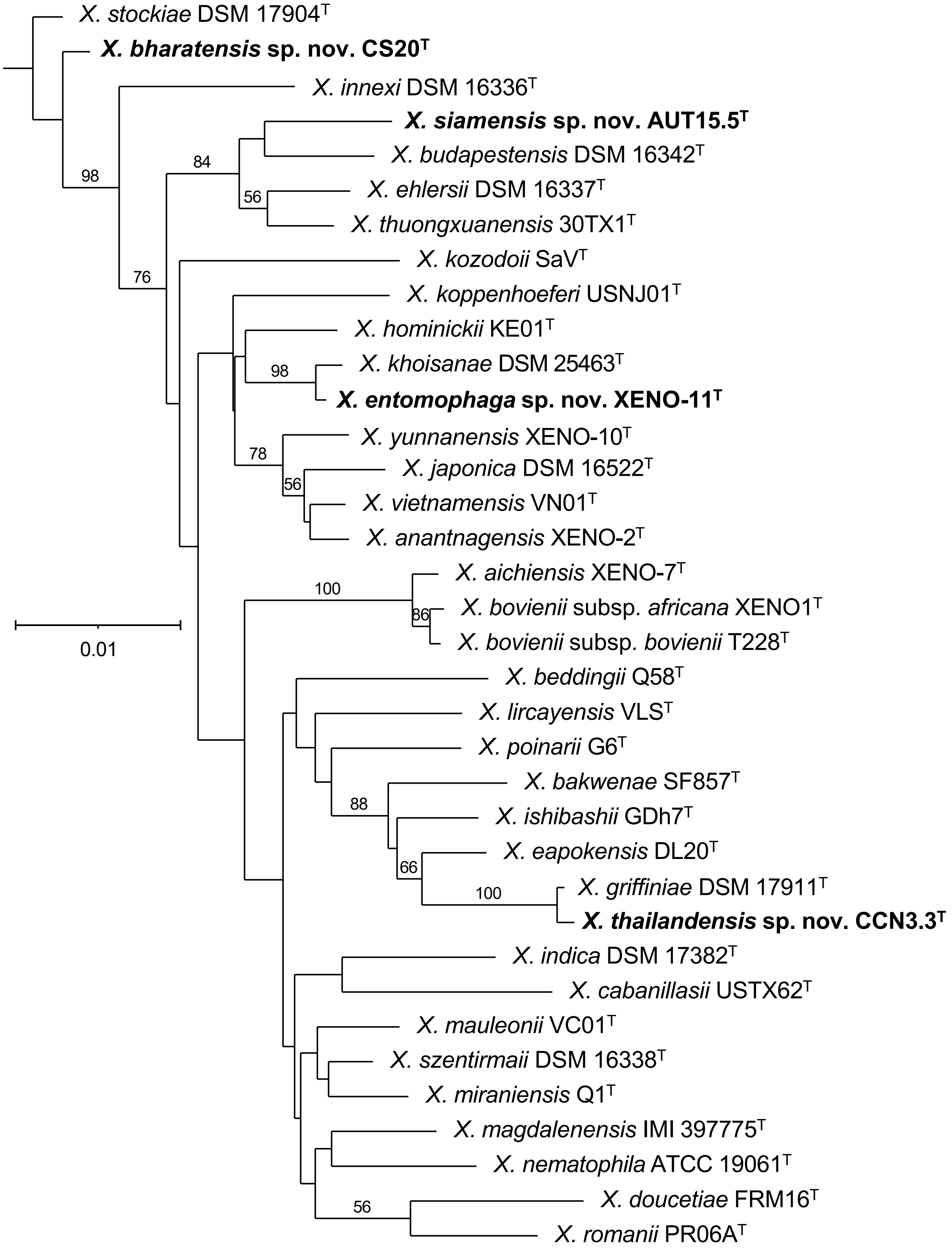

Figure S2. Neighbor-Joining phylogenetic tree reconstructed from the 16S rRNA gene sequences of the type strains of all *Xenorhabdus* species with validly published names. The evolutionary distances were computed using the Kimura 2-parameter model. The percentage of trees in which the associated taxa clustered together is shown next to the branches. The tree is drawn to scale, with branch lengths measured in the number of substitutions per site. There were a total of 1334 positions in the final dataset. Evolutionary analyses were conducted in MEGA7 based on 100 replications. Accession numbers of used gene sequences are shown in Table S1.

	X. stockiae DSM 17904^{T}	***X. bharatensis* sp. nov. CS20^{T}**	*X. innexi* DSM 16336^{T}	***X. siamensis* sp. nov. AUT15.5^{T}**	*X. budapestensis* DSM 16342^{T}	*X. ehlersii* DSM 16337^{T}	*X. thuongxuanensis* 30TX1^{T}	*X. kozodoii* SaV^{T}	*X. koppenhoeferi* USNJ01^{T}	*X. hominickii* KE01^{T}	*X. khoisanae* DSM 25463^{T}	***X. entomophaga* sp. nov. XENO-11^{T}**	*X. yunnanensis* XENO-10^{T}	*X. japonica* DSM 16522^{T}	*X. vietnamensis* VN01^{T}	*X. anantnagensis* XENO-2^{T}	*X. aichiensis* XENO-7^{T}	*X. bovienii* subsp. *africana* XENO-1^{T}	*X. bovienii* subsp. *bovienii* T228^{T}	*X. beddingii* Q58^{T}	*X. lircayensis* VLS^{T}	*X. poinarii* G6^{T}	*X. bakwenae* SF857^{T}	*X. ishibashii* GDh7^{T}	*X. eapokensis* DL20^{T}	*X. griffiniae* DSM 17911^{T}	***X. thailandensis* sp. nov. CCN3.3^{T}**	*X. indica* DSM 17382^{T}	*X. cabanillasii* USTX62^{T}	*X. mauleonii* VC01^{T}	*X. szentirmaii* DSM 16338^{T}	*X. miraniensis* Q1^{T}	*X. magdalenensis* IM139775^{T}	*X. nematophila* ATCC 19061^{T}	*X. doucetiae* FRM16^{T}	*X. romanii* PR6a^{T}
X. stockiae DSM 17904^{T}	ID	97.8	96.7	97.1	97.5	97.5	98.2	96.5	97.1	96.4	96.6	97.1	97.0	96.1	96.5	96.6	96.9	95.9	95.8	96.0	96.4	96.4	96.2	96.3	96.5	96.4	96.1	95.9	95.6	95.9	96.4	96.3	95.7	95.9	96.3	96.2
***X. bharatensis* sp. nov. CS20^{T}**	97.8	ID	96.5	96.2	96.1	96.4	97.1	95.6	95.8	96.2	96.2	96.4	96.4	95.6	95.3	96.1	96.0	95.8	95.6	95.6	97.6	97.5	97.4	97.6	97.2	97.1	96.7	96.8	95.7	97.0	97.2	96.7	96.7	96.4	96.5	96.4
X. innexi DSM 16336^{T}	96.7	96.5	ID	97.3	96.9	98.0	97.8	96.7	96.4	95.9	95.8	96.3	96.6	96.2	96.8	97.2	97.1	96.5	96.5	96.5	97.2	96.7	97.0	96.9	96.9	97.1	96.6	96.7	95.6	95.6	95.6	95.7	95.3	95.9	96.7	96.4
***X. siamensis* sp. nov. AUT15.5^{T}**	97.1	96.2	97.3	ID	98.1	98.0	97.4	97.1	97.7	96.2	96.2	97.2	97.1	97.2	96.7	96.9	96.9	96.9	96.7	97.0	96.7	96.9	96.6	96.6	96.7	96.6	96.5	96.3	95.8	95.8	96.1	96.1	95.8	95.8	97.2	96.8
X. budapestensis DSM 16342^{T}	97.5	96.1	96.9	98.1	ID	98.5	98	97.5	97.5	96.7	96.7	97.8	97.6	97.2	97.0	97.1	97.5	96.8	96.7	97.1	96.9	97	96.6	96.9	97.6	97.5	97	96.5	96.2	96.9	97.1	96.8	96.6	96.4	97.2	96.9
X. ehlersii DSM 16337^{T}	97.5	96.4	98.0	98.0	98.5	ID	98.8	97.8	97.9	96.7	96.7	97.4	97.8	97.5	97.4	98.0	97.5	97.2	97.1	97.2	97.5	97.2	97.5	97.5	97.5	97.6	97.3	97.7	96.2	96.6	96.5	96.5	96.5	96.8	97.5	97.3
X. thuongxuanensis 30TX1^{T}	98.2	97.1	97.8	97.4	98.0	98.8	ID	97.1	97.3	97.1	97.2	97.5	97.5	97.2	97.2	97.8	97.2	97.2	97.0	97.2	97.3	97.2	97.2	96.9	97.1	96.9	97.2	97.0	96.1	96.3	96.6	96.2	96.1	96.8	97.3	97.2
X. kozodoii SaV^{T}	96.5	95.6	96.7	97.1	97.5	97.8	97.1	ID	97.5	95.7	95.6	96.7	96.9	96.3	96.4	96.6	96.9	96.1	96.0	96.0	95.8	95.7	96.2	96.0	96.1	95.8	95.8	96.3	94.8	95.8	95.7	96.1	96.1	96.1	96.6	96.3
X. koppenhoeferi USNJ01^{T}	97.1	95.8	96.4	97.7	97.5	97.9	97.3	97.5	ID	96.5	96.5	97.5	97.7	96.9	97.0	97.0	96.9	96.1	96.0	96.4	96.7	96.5	96.6	96.4	96.7	96.7	96.9	96.8	96.5	96.4	96.7	96.8	97.5	96.7	97.7	97.3
X. hominickii KE01^{T}	96.4	96.2	95.9	96.2	96.7	96.7	97.1	95.7	96.5	ID	99.7	97.6	97.9	97.8	96.7	96.8	95.9	95.8	95.7	95.9	95.8	96.1	95.6	95.6	96.5	96.4	96.0	95.6	96.1	96.5	96.9	96.1	95.9	96	96.4	96.0
X. khoisanae DSM 25463^{T}	96.6	96.2	95.8	96.2	96.7	96.7	97.2	95.6	96.5	99.7	ID	97.8	97.9	98.1	96.7	96.8	96.1	95.8	95.8	95.9	96.0	96.1	95.4	95.5	96.4	96.3	95.8	95.4	96.3	96.4	96.9	96.0	95.8	95.8	96.2	95.8
***X. entomophaga* sp. nov. XENO-11^{T}**	97.1	96.4	96.3	97.2	97.8	97.4	97.5	96.7	97.5	97.6	97.8	ID	98.6	97.8	97.7	97.2	96.9	96.3	96.2	96.6	96.2	96.0	96.1	96.2	97.1	96.9	97.0	96	96.1	97.2	97.5	96.3	96.9	96.5	97.2	96.8
X. yunnanensis XENO-10^{T}	97.0	96.4	96.6	97.1	97.6	97.8	97.5	96.9	97.7	97.9	97.9	98.6	ID	98.3	97.7	97.2	96.7	96.5	96.5	96.8	96.1	96.1	96.1	96.1	96.9	96.9	96.9	96.3	96.5	97.6	97.6	96.6	97.1	96.7	97.1	96.7
X. japonica DSM 16522^{T}	96.1	95.6	96.2	97.2	97.2	97.5	97.2	96.3	96.9	97.8	98.1	97.8	98.3	ID	96.9	97.1	96.3	96.5	96.4	96.5	96.1	96.2	96.0	95.8	96.6	96.5	96.3	96.1	96.6	96.8	97.0	96.4	96.4	96.1	96.7	96.1
X. vietnamensis VN01^{T}	96.5	95.3	96.8	96.7	97.0	97.4	97.2	96.4	97.0	96.7	96.7	97.7	97.7	96.9	ID	97.3	97.5	96.7	96.6	96.8	96.1	96.1	96.2	95.9	96.5	96.4	96.6	96.1	96.1	96.4	96.3	95.8	95.8	96.1	96.8	96.5
X. anantnagensis XENO-2^{T}	96.6	96.1	97.2	96.9	97.1	98.0	97.8	96.6	97.0	96.8	96.8	97.2	97.2	97.1	97.3	ID	96.9	97.0	96.7	96.9	96.8	96.6	96.8	97.3	97.7	97.5	97.3	97.1	96.3	95.9	95.6	95.7	95.7	95.7	96.4	96.1
X. aichiensis XENO-7^{T}	96.9	96.0	97.1	96.9	97.5	97.5	97.2	96.9	96.9	95.9	96.1	96.9	96.7	96.3	97.5	96.9	ID	96.1	96.1	96.2	96.7	96.4	96.4	96.4	96.7	96.7	96.3	96.1	95.8	95.8	95.9	95.7	95.3	96.1	96.7	96.4
X. bovienii subsp. *africana* XENO-1^{T}	95.9	95.8	96.5	96.9	96.8	97.2	97.2	96.1	96.1	95.8	95.8	96.3	96.5	96.5	96.7	97.0	96.1	ID	99.4	99.4	96.6	96.9	96.9	96.7	96.8	96.9	97.0	96.8	95.8	96.1	96.1	96.3	95.9	95.9	96.4	96.1
X. bovienii subsp. *bovienii* T228^{T}	95.8	95.6	96.5	96.7	96.7	97.1	97.0	96.0	96.0	95.7	95.8	96.2	96.5	96.4	96.6	96.7	96.1	99.4	ID	99.2	96.6	96.7	96.7	96.4	96.7	96.9	96.8	96.4	95.8	96.3	96.1	96.3	95.8	96.0	96.2	96.0
X. beddingii Q58^{T}	96.0	95.6	96.5	97.0	97.1	97.2	97.2	96.0	96.4	95.9	95.9	96.6	96.8	96.5	96.8	96.9	96.2	99.4	99.2	ID	96.7	96.7	96.9	96.7	97.2	97.2	97.5	96.6	96.2	96.4	96.4	96.3	96.3	96.2	96.7	96.4
X. lircayensis VLS^{T}	96.4	97.6	97.2	96.7	96.9	97.5	97.3	95.8	96.7	95.8	96.0	96.2	96.1	96.1	96.1	96.8	96.7	96.6	96.6	96.7	ID	98.9	98.9	98.7	98.1	98.2	97.6	97.7	97.1	97.3	97.5	96.9	96.7	97.2	97.5	97.3
X. poinarii G6^{T}	96.4	97.5	96.7	96.9	97.0	97.2	97.2	95.7	96.5	96.1	96.1	96.0	96.1	96.2	96.1	96.6	96.4	96.9	96.7	96.7	98.9	ID	98.7	98.3	98.0	97.8	97.5	97.4	96.7	97.2	97.6	96.9	96.6	97.1	97.5	97.2
X. bakwenae SF857^{T}	96.2	97.4	97.0	96.6	96.6	97.5	97.2	96.2	96.6	95.6	95.4	96.1	96.1	96.0	96.2	96.8	96.4	96.9	96.7	96.9	98.9	98.7	ID	98.3	97.8	97.7	97.4	97.8	96.5	96.9	97.1	96.9	96.9	97.3	97.3	97.0
X. ishibashii GDh7^{T}	96.3	97.6	96.9	96.6	96.9	97.5	96.9	96.0	96.4	95.6	95.5	96.2	96.1	95.8	95.9	97.3	96.4	96.7	96.4	96.7	98.7	98.3	98.3	ID	98.5	98.4	98.0	97.8	96.6	97.2	96.9	96.9	96.7	96.7	97.1	96.9
X. eapokensis DL20^{T}	96.5	97.2	96.9	96.7	97.6	97.5	97.1	96.1	96.7	96.5	96.4	97.1	96.9	96.6	96.5	97.7	96.7	96.8	96.7	97.2	98.1	98.0	97.8	98.5	ID	99.6	98.6	97.8	97.2	97.6	97.5	97.2	97.3	97.2	97.5	97.4
X. griffiniae DSM 17911^{T}	96.4	97.1	97.1	96.6	97.5	97.6	96.9	95.8	96.7	96.4	96.3	96.9	96.9	96.5	96.4	97.5	96.7	96.9	96.9	97.2	98.2	97.8	97.7	98.4	99.6	ID	98.6	97.8	97.5	97.6	97.3	97.2	97.2	97.2	97.5	97.3
***X. thailandensis* sp. nov. CCN3.3^{T}**	96.1	96.7	96.6	96.5	97.0	97.3	97.2	95.8	96.9	96.0	95.8	97.0	96.9	96.3	96.6	97.3	96.3	97.0	96.8	97.5	97.6	97.5	97.4	98.0	98.6	98.6	ID	97.7	96.7	97.3	97.3	97.0	97.3	97.3	97.8	97.6
X. indica DSM 17382^{T}	95.9	96.8	96.7	96.3	96.5	97.7	97.0	96.3	96.8	95.6	95.4	96.0	96.3	96.1	96.1	97.1	96.1	96.8	96.4	96.6	97.7	97.4	97.8	97.8	97.8	97.8	97.7	ID	96.4	96.8	96.7	96.9	96.9	96.7	97.1	96.8
X. cabanillasii USTX62^{T}	95.6	95.7	95.6	95.8	96.2	96.2	96.1	94.8	96.5	96.1	96.3	96.1	96.5	96.6	96.1	96.3	95.8	95.8	95.8	96.2	97.1	96.7	96.5	96.6	97.2	97.5	96.7	96.4	ID	96.6	96.8	96.1	96.1	96.4	97.3	96.9
X. mauleonii VC01^{T}	95.9	97.0	95.6	95.8	96.9	96.6	96.3	95.8	96.4	96.5	96.4	97.2	97.6	96.8	96.4	95.9	95.8	96.1	96.3	96.4	97.3	97.2	96.9	97.2	97.6	97.6	97.3	96.8	96.6	ID	98.2	97.4	97.8	97.2	97.0	96.9
X. szentirmaii DSM 16338^{T}	96.4	97.2	95.6	96.1	97.1	96.5	96.6	95.7	96.7	96.9	96.9	97.5	97.6	97.0	96.3	95.6	95.9	96.1	96.1	96.4	97.5	97.6	97.1	96.9	97.5	97.3	97.3	96.7	96.8	98.2	ID	98.1	97.7	97.7	97.5	97.4
X. miraniensis Q1^{T}	96.3	96.7	95.7	96.1	96.8	96.5	96.2	96.1	96.8	96.1	96	96.3	96.6	96.4	95.8	95.7	95.7	96.3	96.3	96.3	96.9	96.9	96.9	96.9	97.2	97.2	97.0	96.9	96.1	97.4	98.1	ID	97.8	97.3	97.2	97.2
X. magdalenensis IM139775^{T}	95.7	96.7	95.3	95.8	96.6	96.5	96.1	96.1	97.5	95.9	95.8	96.9	97.1	96.4	95.8	95.7	95.3	95.9	95.8	96.3	96.7	96.6	96.9	96.7	97.3	97.2	97.3	96.9	96.1	97.8	97.7	97.8	ID	96.8	96.8	96.7
X. nematophila ATCC 19061^{T}	95.9	96.4	95.9	95.8	96.4	96.8	96.8	96.1	96.7	96.0	95.8	96.5	96.7	96.1	96.1	95.7	96.1	95.9	96.0	96.2	97.2	97.1	97.3	96.7	97.2	97.2	97.3	96.7	96.4	97.2	97.7	97.3	96.8	ID	98.2	98.0
X. doucetiae FRM16^{T}	96.3	96.5	96.7	97.2	97.2	97.5	97.3	96.6	97.7	96.4	96.2	97.2	97.1	96.7	96.8	96.4	96.7	96.4	96.2	96.7	97.5	97.5	97.3	97.1	97.5	97.5	97.8	97.1	97.3	97.0	97.5	97.2	96.8	98.2	ID	99.4
X. romanii PR6a^{T}	96.2	96.4	96.4	96.8	96.9	97.3	97.2	96.3	97.3	96.0	95.8	96.8	96.7	96.1	96.5	96.1	96.4	96.1	96	96.4	97.3	97.2	97.0	96.9	97.4	97.3	97.6	96.8	96.9	96.9	97.4	97.2	96.7	98.0	99.4	ID

Figure S3. Pairwise comparisons of the nucleotide similarity values (%) of the 16S rRNA gene sequences of the type strains of all *Xenorhabdus* species with validly published names. A total of 1334 nucleotide positions were analyzed. Accession numbers of used gene sequences are shown in Table S1.

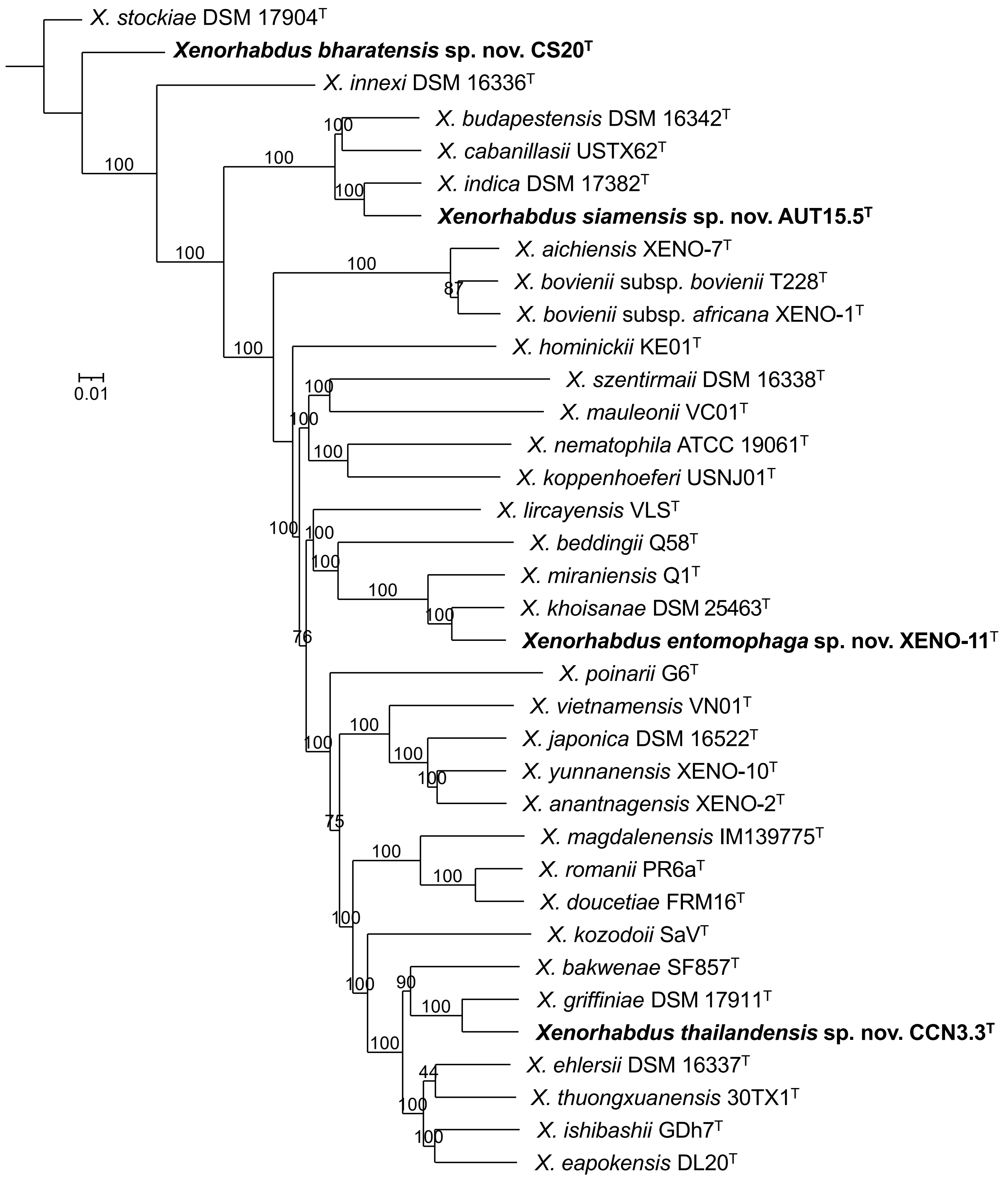

Figure S4. Phylogenetic reconstruction based on whole genome sequences of the type strains of all *Xenorhabdus* species with validly published names. Trees were inferred with FastME 2.1.6.1 from GBDP distances calculated from genome sequences. The branch lengths are scaled in terms of GBDP distance formula d_5. The numbers above branches are GBDP pseudo-bootstrap support values from 100 replications. NCBI accession numbers of the sequences used for the analyses are shown in Table S1.

	X. stockiae DSM 17904^{T}	***Xenorhabdus bharatensis* sp. nov. CS20^{T}**	*X. innexi* DSM 16336^{T}	*X. budapestensis* DSM 16342^{T}	*X. cabanillasii* USTX62^{T}	*X. indica* DSM 17382^{T}	***X. siamensis* sp. nov. AUT15.5^{T}**	*X. aichiensis* XENO-7^{T}	*X. bovienii* subsp. *bovienii* T228^{T}	*X. bovienii* subsp. *africana* XENO-1^{T}	*X. hominickii* KE01^{T}	*X. szentirmaii* DSM 16338^{T}	*X. mauleonii* VC01^{T}	*X. nematophila* ATCC 19061^{T}	*X. koppenhoeferi* USNJ01^{T}	*X. lircayensis* VLS^{T}	*X. beddingii* Q58^{T}	*X. miraniensis* Q1^{T}
X. stockiae DSM 17904^{T}	ID	93.1	93.2	92.2	89.9	90.0	89.6	87.3	86.4	86.5	86.5	86.5	86.5	86.4	86.1	83.9	84.2	84.2
***Xenorhabdus bharatensis* sp. nov. CS20^{T}**	93.1	ID	92.2	91.7	89.5	89.7	89.5	87.1	86.3	86.4	86.4	86.5	86.3	86.5	86.2	84.1	84.2	84.1
X. innexi DSM 16336^{T}	92.9	92.1	ID	93.4	90.8	90.6	90.1	87.3	86.1	86.3	86.6	86.7	86.8	86.6	86.3	83.7	84.0	84.1
X. budapestensis DSM 16342^{T}	92.2	91.7	93.4	ID	91.4	91.9	91.5	87.8	86.7	87.0	87.0	87.1	87.1	87.1	86.7	84.0	84.5	84.6
X. cabanillasii USTX62^{T}	89.9	89.6	90.7	91.4	ID	94.9	90.8	87.2	86.2	86.3	86.3	86.1	86.1	86.1	85.7	83.8	84.0	84.0
X. indica DSM 17382^{T}	90.0	89.6	90.5	91.9	94.9	ID	90.8	87.0	85.9	86.2	86.3	86.2	86.4	86.1	85.7	83.6	84.1	84.0
***X. siamensis* sp. nov. AUT15.5^{T}**	89.6	89.4	90.3	91.5	90.9	90.9	ID	87.4	86.5	86.6	86.7	86.2	86.3	86.1	85.8	83.8	84.6	84.9
X. aichiensis XENO-7^{T}	87.1	87.0	87.3	87.7	87.2	87.0	87.3	ID	85.8	85.9	86.3	85.7	85.7	85.4	85.1	83.5	83.6	83.4
X. bovienii subsp. *bovienii* T228^{T}	86.2	86.3	86.2	86.7	86.2	85.9	86.4	85.9	ID	96.3	91.7	85.6	85.5	85.5	85.2	84.1	83.5	83.5
X. bovienii subsp. *africana* XENO-1^{T}	86.4	86.4	86.5	86.9	86.3	86.2	86.6	86.1	96.3	ID	91.9	85.9	85.8	85.7	85.4	84.2	83.7	83.7
X. hominickii KE01^{T}	86.5	86.5	86.6	87.0	86.4	86.4	86.6	86.3	91.8	91.9	ID	85.9	86.1	85.8	85.4	84.1	83.7	83.8
X. szentirmaii DSM 16338^{T}	86.5	86.5	86.7	87.1	86.1	86.1	86.1	85.5	85.4	85.7	85.9	ID	94.4	93.1	90.4	84.7	84.8	85.0
X. mauleonii VC01^{T}	86.4	86.3	86.7	87.0	86.1	86.3	86.3	85.6	85.4	85.7	86.0	94.4	ID	93.4	90.5	84.8	84.7	84.8
X. nematophila ATCC 19061^{T}	86.3	86.5	86.8	87.0	86.0	86.1	86.1	85.3	85.5	85.7	85.8	93.3	93.7	ID	90.7	84.8	84.6	84.7
X. koppenhoeferi USNJ01^{T}	86.1	86.0	86.2	86.5	85.8	85.8	85.8	85.1	85.1	85.3	85.6	90.4	90.6	90.5	ID	84.6	84.4	84.3
X. lircayensis VLS^{T}	83.7	84.0	83.9	84.1	83.8	83.6	83.8	83.5	84.1	84.0	84.1	84.9	85.0	84.8	84.8	ID	82.5	82.6
X. beddingii Q58^{T}	84.0	84.0	84.1	84.2	83.6	83.8	84.4	83.6	83.3	83.5	83.4	84.6	84.4	84.4	84.3	82.5	ID	95.5
X. miraniensis Q1^{T}	84.1	84.0	84.3	84.5	83.7	84.0	84.7	83.3	83.4	83.7	83.7	85.0	84.9	84.5	84.5	82.4	95.6	ID
X. khoisanae DSM 25463^{T}	84.1	84.2	84.1	84.4	83.8	83.7	84.1	83.6	83.4	83.6	83.7	84.8	84.7	84.9	84.5	82.7	93.5	93.4
***X. entomophaga* sp. nov. XENO-11^{T}**	83.8	83.8	83.7	83.9	83.5	83.4	83.7	83.3	83.7	83.7	83.8	84.0	84.0	84.0	83.9	82.4	86.1	86.2
X. poinarii G6^{T}	84.3	84.6	84.8	84.8	84.3	84.3	84.4	83.8	84.4	84.5	84.8	85.3	85.2	85.1	85.0	83.1	85.6	85.8
X. vietnamensis VN01^{T}	83.1	83.2	83.1	83.3	82.8	83.0	83.2	82.4	83.3	83.2	83.1	83.8	83.6	83.6	83.4	82.3	84.0	83.9
X. japonica DSM 16522^{T}	83.0	83.1	82.8	82.9	82.8	82.7	82.9	82.5	83.1	83.1	83.1	83.2	83.2	83.3	83.2	82.5	83.5	83.5
X. yunnanensis XENO-10^{T}	82.0	82.2	82.2	82.3	82.2	82.0	82.1	81.9	82.1	82.3	82.1	82.5	82.4	82.5	82.2	81.8	82.6	82.5
X. anantnagensis XENO-2^{T}	81.9	82.0	81.8	82.0	81.7	81.7	81.9	81.5	82.1	82.4	81.9	81.9	82.0	82.1	81.9	81.6	82.2	82.1
X. magdalenensis IM139775^{T}	82.9	83.4	83.1	83.5	82.8	82.7	82.9	82.6	82.9	82.6	82.7	84.1	84.1	83.8	84.0	82.1	83.7	83.8
X. romanii PR6a^{T}	82.0	82.0	81.8	82.3	81.7	81.8	82.2	81.7	81.8	82.0	82.0	82.5	82.3	82.4	82.1	81.5	82.5	82.8
X. doucetiae FRM16^{T}	82.0	82.1	81.9	82.3	82.0	82.0	81.9	82.0	81.9	82.1	82.1	82.5	82.4	82.3	82.3	81.5	82.6	82.6
X. kozodoii SaV^{T}	82.1	82.2	81.9	82.3	81.8	82.1	82.0	81.9	81.8	81.9	81.9	82.6	82.5	82.6	82.3	81.4	82.5	82.6
X. bakwenae SF857^{T}	81.9	82.0	81.8	81.9	82.0	82.0	82.1	81.5	81.2	81.1	81.1	81.8	81.8	81.8	81.6	81.2	81.9	81.9
X. griffiniae DSM 17911^{T}	81.9	81.9	81.5	81.8	81.7	81.8	81.6	81.6	80.9	81.2	80.9	81.9	81.8	81.9	81.7	81.0	82.0	81.9
***X. thailandensis* sp. nov. CCN3.3^{T}**	81.5	81.6	81.5	81.8	81.4	81.3	81.3	81.1	81.4	81.4	81.4	81.6	81.6	81.7	81.7	81.5	81.7	81.9
X. ehlersii DSM 16337^{T}	82.1	81.8	81.9	82.2	81.6	81.9	81.9	81.3	81.2	81.3	81.1	81.9	82.1	81.9	81.8	81.1	82.0	82.0
X. thuongxuanensis 30TX1^{T}	81.1	81.0	80.9	81.1	80.9	80.9	81.0	80.7	81.1	81.3	81.1	81.2	81.0	81.1	81.0	81.1	81.1	81.0
X. ishibashii GDh7^{T}	80.9	80.9	80.8	81.1	81.0	81.1	81.0	80.5	80.9	81.0	80.8	81.3	81.1	81.0	81.0	80.7	81.0	81.1
X. eapokensis DL20^{T}	81.2	81.1	81.0	81.3	81.1	81.0	81.0	80.8	81.0	81.0	80.8	81.3	81.3	81.4	81.4	80.8	81.3	81.1

	X. khoisanae DSM 25463^{T}	***X. entomophaga* sp. nov. XENO-11^{T}**	*X. poinarii* G6^{T}	*X. vietnamensis* VN01^{T}	*X. japonica* DSM 16522^{T}	*X. yunnanensis* XENO-10^{T}	*X. anantnagensis* XENO-2^{T}	*X. magdalenensis* IM139775^{T}	*X. romanii* PR6a^{T}	*X. doucetiae* FRM16^{T}	*X. kozodoii* SaV^{T}	*X. bakwenae* SF857^{T}	*X. griffiniae* DSM 17911^{T}	***X. thailandensis* sp. nov. CCN3.3^{T}**	*X. ehlersii* DSM 16337^{T}	*X. thuongxuanensis* 30TX1^{T}	*X. ishibashii* GDh7^{T}	*X. eapokensis* DL20^{T}
X. stockiae DSM 17904^{T}	84.2	83.9	84.6	83.1	83.0	82.4	81.9	83.2	82.2	82.2	82.2	81.9	82.1	81.5	81.9	81.0	81.0	81.2
***Xenorhabdus bharatensis* sp. nov. CS20^{T}**	84.3	83.7	84.8	83.1	83.1	82.4	82.0	83.3	82.1	82.1	82.4	82.1	82.0	81.6	81.8	81.1	81.1	81.3
X. innexi DSM 16336^{T}	84.2	83.7	84.6	83.2	82.7	82.1	81.8	83.1	81.8	82.0	82.0	81.8	81.6	81.5	81.9	80.8	80.7	81.0
X. budapestensis DSM 16342^{T}	84.6	84.0	85.0	83.5	83.1	82.4	82.2	83.6	82.4	82.5	82.4	82.0	81.9	81.7	82.2	81.0	81.3	81.4
X. cabanillasii USTX62^{T}	83.9	83.6	84.5	83.0	82.7	82.2	81.9	82.9	81.9	81.9	82.0	81.9	81.8	81.6	81.6	81.1	80.9	81.0
X. indica DSM 17382^{T}	83.9	83.4	84.4	82.8	82.8	82.1	81.8	82.8	82.0	82.0	82.1	81.9	81.8	81.4	81.9	80.7	81.1	81.1
***X. siamensis* sp. nov. AUT15.5^{T}**	84.2	83.6	84.4	83.1	82.9	82.0	82.0	83.0	82.3	81.9	81.9	81.8	81.7	81.3	81.8	80.9	80.7	81.1
X. aichiensis XENO-7^{T}	83.8	83.4	84.0	82.6	82.4	82.0	81.6	82.6	81.9	82.0	82.1	81.3	81.7	81.2	81.2	80.7	80.6	80.8
X. bovienii subsp. *bovienii* T228^{T}	83.6	83.8	84.5	83.2	83.1	82.4	82.1	82.9	82.0	82.1	82.0	81.3	81.2	81.4	81.2	81.1	80.9	81.1
X. bovienii subsp. *africana* XENO-1^{T}	83.8	83.8	84.7	83.1	82.9	82.4	82.4	82.8	82.1	82.1	82.1	81.2	81.1	81.4	81.3	81.3	81.0	81.0
X. hominickii KE01^{T}	83.7	83.7	84.9	83.1	83.1	82.3	82.1	82.9	81.9	82.0	82.1	81.2	81.1	81.3	81.1	81.1	80.9	81.0
X. szentirmaii DSM 16338^{T}	84.8	84.2	85.5	83.6	83.2	82.5	81.9	84.1	82.5	82.5	82.6	81.7	81.7	81.6	81.9	81.1	81.1	81.4
X. mauleonii VC01^{T}	84.9	84.1	85.5	83.6	83.3	82.3	82.0	84.1	82.5	82.4	82.7	81.6	81.8	81.5	82.2	80.9	81.2	81.3
X. nematophila ATCC 19061^{T}	84.9	84.0	85.3	83.6	83.2	82.6	82.2	83.7	82.2	82.1	82.4	81.8	81.8	81.5	81.8	81.0	81.1	81.3
X. koppenhoeferi USNJ01^{T}	84.5	84.0	85.1	83.3	83.1	82.4	81.9	83.8	82.2	82.2	82.3	81.6	81.7	81.5	81.8	81.0	80.9	81.3
X. lircayensis VLS^{T}	82.8	82.5	83.1	82.3	82.7	81.8	81.6	82.2	81.5	81.4	81.7	81.2	81.1	81.5	81.2	81.1	80.8	80.9
X. beddingii Q58^{T}	93.3	86.1	85.6	83.7	83.5	82.6	82.1	83.6	82.5	82.4	82.5	81.8	82.0	81.7	81.8	80.9	80.9	81.2
X. miraniensis Q1^{T}	93.4	86.2	85.8	83.8	83.3	82.4	82.2	83.8	82.7	82.6	82.6	81.7	81.8	81.8	82.1	81.0	81.2	81.2
X. khoisanae DSM 25463^{T}	ID	86.5	85.7	83.8	83.4	82.8	82.4	83.8	82.5	82.5	82.7	82.0	82.2	81.9	82.0	81.2	81.2	81.6
***X. entomophaga* sp. nov. XENO-11^{T}**	86.4	ID	85.4	83.4	83.2	82.5	82.2	83.3	82.2	82.3	82.4	81.6	81.6	81.4	81.3	80.9	81.0	81.0
X. poinarii G6^{T}	85.6	85.3	ID	85.5	84.7	83.5	83.0	84.9	83.0	83.2	83.3	81.5	81.4	82.0	81.6	81.4	81.1	81.1
X. vietnamensis VN01^{T}	83.9	83.5	85.5	ID	87.3	83.5	82.7	83.3	82.2	82.4	82.5	81.4	81.4	81.8	81.5	81.2	80.9	81.0
X. japonica DSM 16522^{T}	83.5	83.2	84.9	87.1	ID	83.4	83.4	82.8	82.0	82.2	82.2	81.7	81.4	82.5	81.7	81.6	80.9	81.2
X. yunnanensis XENO-10^{T}	82.5	82.5	83.4	83.3	83.2	ID	83.5	82.1	81.5	81.4	81.5	81.1	81.0	81.7	81.1	81.2	80.8	80.8
X. anantnagensis XENO-2^{T}	82.3	82.2	82.9	82.5	83.2	83.5	ID	81.7	81.2	81.2	81.3	80.6	80.5	81.2	80.9	81.0	80.5	80.6
X. magdalenensis IM139775^{T}	83.7	83.3	84.9	83.1	82.9	82.2	81.9	ID	82.8	83.0	82.7	81.7	81.9	81.6	82.0	81.4	81.7	81.5
X. romanii PR6a^{T}	82.5	82.3	83.1	82.1	81.9	81.5	81.3	82.8	ID	96.3	95.3	81.9	82.0	81.8	82.1	81.2	81.6	81.5
X. doucetiae FRM16^{T}	82.5	82.3	83.3	82.2	82.1	81.5	81.3	83.0	96.4	ID	96.2	81.8	81.7	82.0	82.0	81.5	81.5	81.7
X. kozodoii SaV^{T}	82.6	82.4	83.2	82.4	82.1	81.5	81.3	82.7	95.4	96.2	ID	81.7	81.9	82.0	82.1	81.4	81.5	81.4
X. bakwenae SF857^{T}	82.1	81.6	81.6	81.4	81.5	80.9	80.9	81.8	82.1	81.7	81.8	ID	95.1	92.8	92.8	83.4	83.6	83.8
X. griffiniae DSM 17911^{T}	82.1	81.4	81.5	81.3	81.2	81.0	80.6	81.8	81.9	81.6	81.8	95.1	ID	92.7	92.7	83.4	83.5	83.8
***X. thailandensis* sp. nov. CCN3.3^{T}**	81.9	81.5	82.0	81.8	82.4	81.8	81.2	81.5	81.8	81.9	82.0	92.7	92.7	ID	93.3	84.0	83.6	83.7
X. ehlersii DSM 16337^{T}	82.1	81.3	81.7	81.5	81.4	81.2	81.1	82.1	82.2	82.0	82.0	92.9	92.8	93.3	ID	83.4	83.7	83.9
X. thuongxuanensis 30TX1^{T}	81.0	81.0	81.4	81.2	81.6	81.3	81.3	81.3	81.4	81.5	81.4	83.2	83.2	83.9	83.4	ID	87.4	87.6
X. ishibashii GDh7^{T}	81.2	80.8	81.1	81.1	81.0	80.6	80.6	81.7	81.7	81.6	81.4	83.5	83.3	83.7	83.7	87.2	ID	93.4
X. eapokensis DL20^{T}	81.4	81.1	81.2	80.9	81.1	80.8	80.5	81.6	81.4	81.4	81.3	83.5	83.7	83.6	83.8	87.5	93.5	ID

Figure S5. Pairwise comparisons of average nucleotide identity (ANI) values (%) of the type strains of all *Xenorhabdus* species with validly published names. Accession numbers of used gene sequences are shown in Table S1.

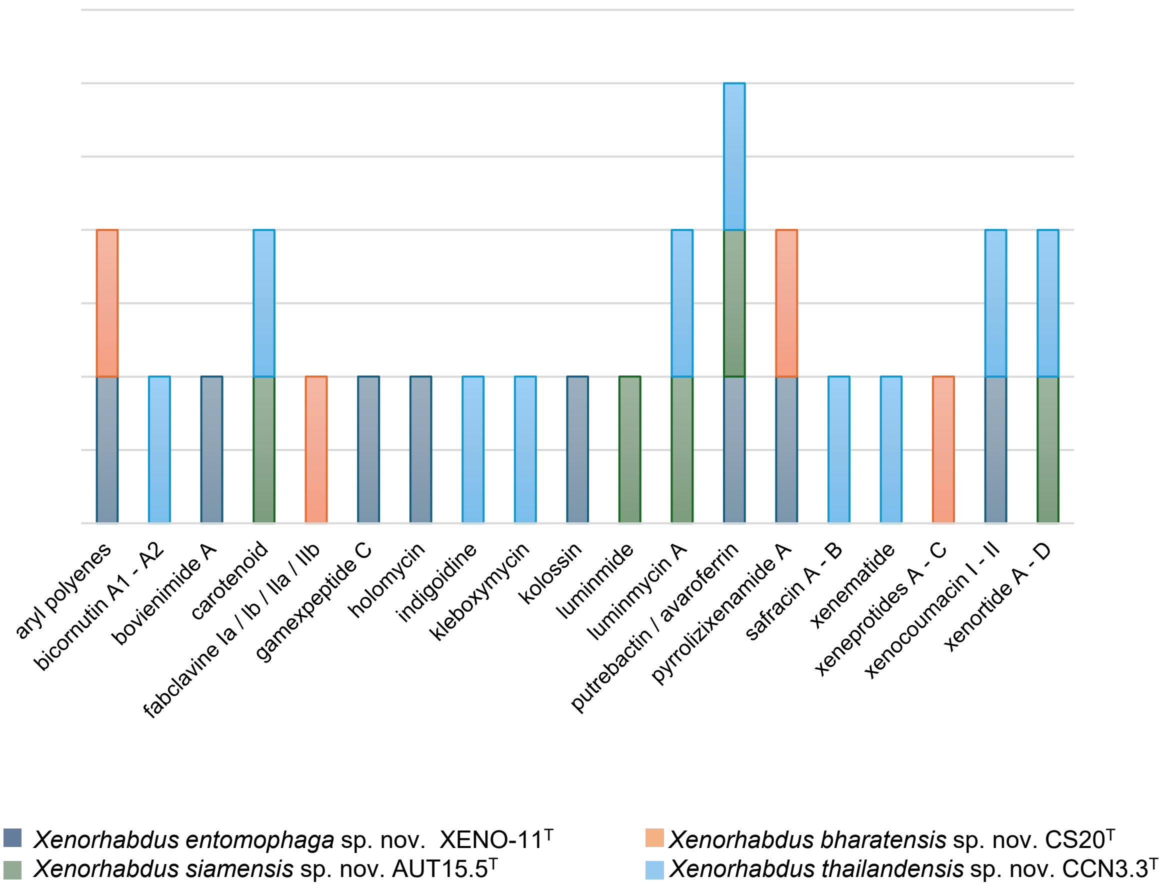

Figure S6. Predicted secondary metabolites produced by *Xenorhabdus bharatensis* sp. nov. CS20^{T}, *X. entomophaga* sp. nov. XENO-11^{T}, *X. siamensis* sp. nov. AUT15.5^{T}, and *X. thailandensis* sp. nov. CCN3.3^{T}.

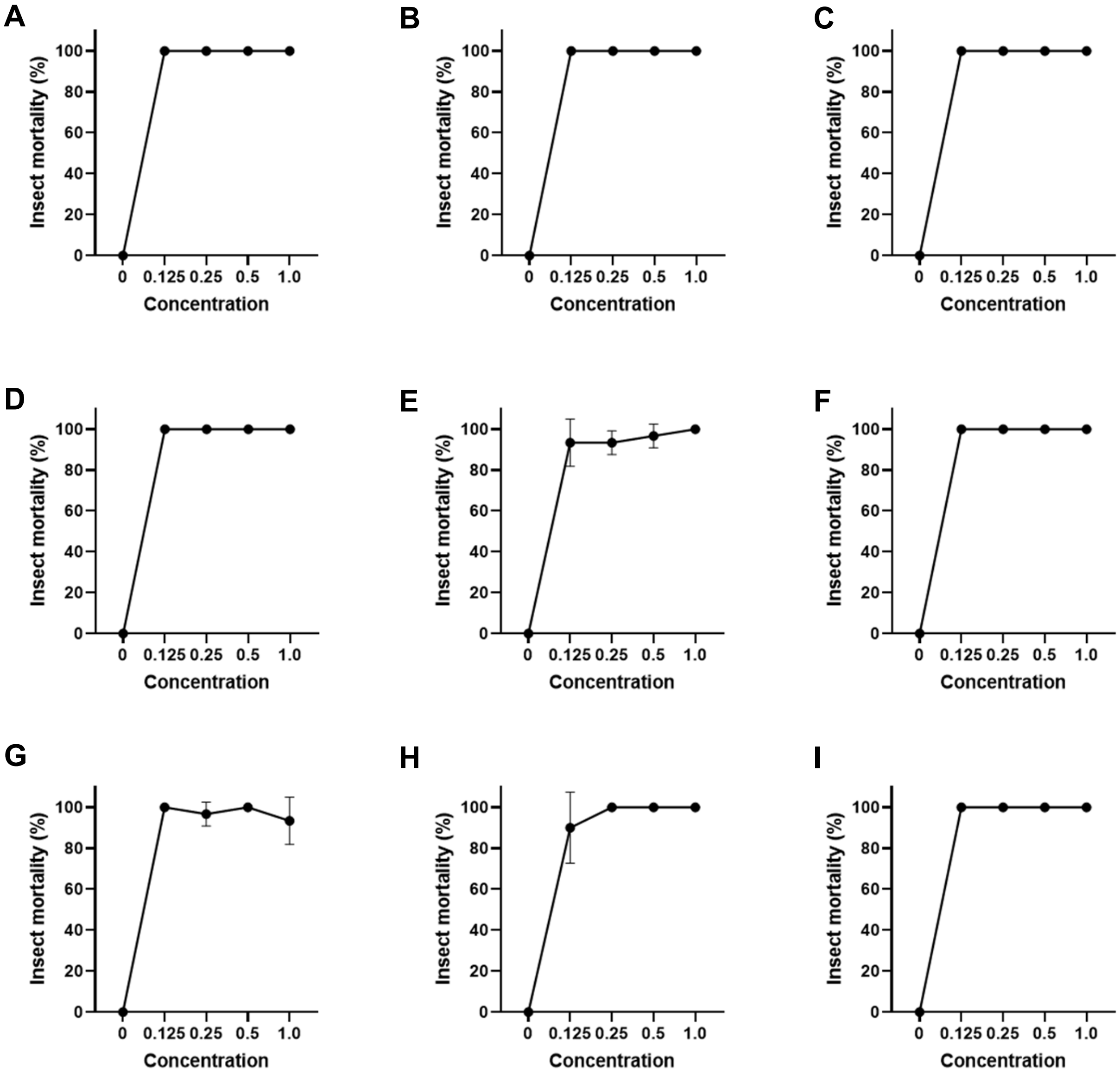

Figure S7. Mortality rate (%) of waxworm larvae 24h after injecting different *Xenorhabdus* strains at various cell concentrations (OD_{600} = 0.125, 0.25, 0.5 and 1.0). A) *X. bharatensis* sp. nov. CS20^T B) *X. entomophaga* sp. nov. XENO-11^T; C) *X. griffiniae* DSM 17911^T; D) *X. indica* DSM 17382^T; E) *X. innexi* DSM 16336^T; F) *X. khoisanae* DSM 25463^T; G) *X. siamensis* sp. nov. AUT15.5^T; H) *X. stockiae* DSM 17904^T; I) *X. thailandensis* sp. nov. CCN3.3^T.

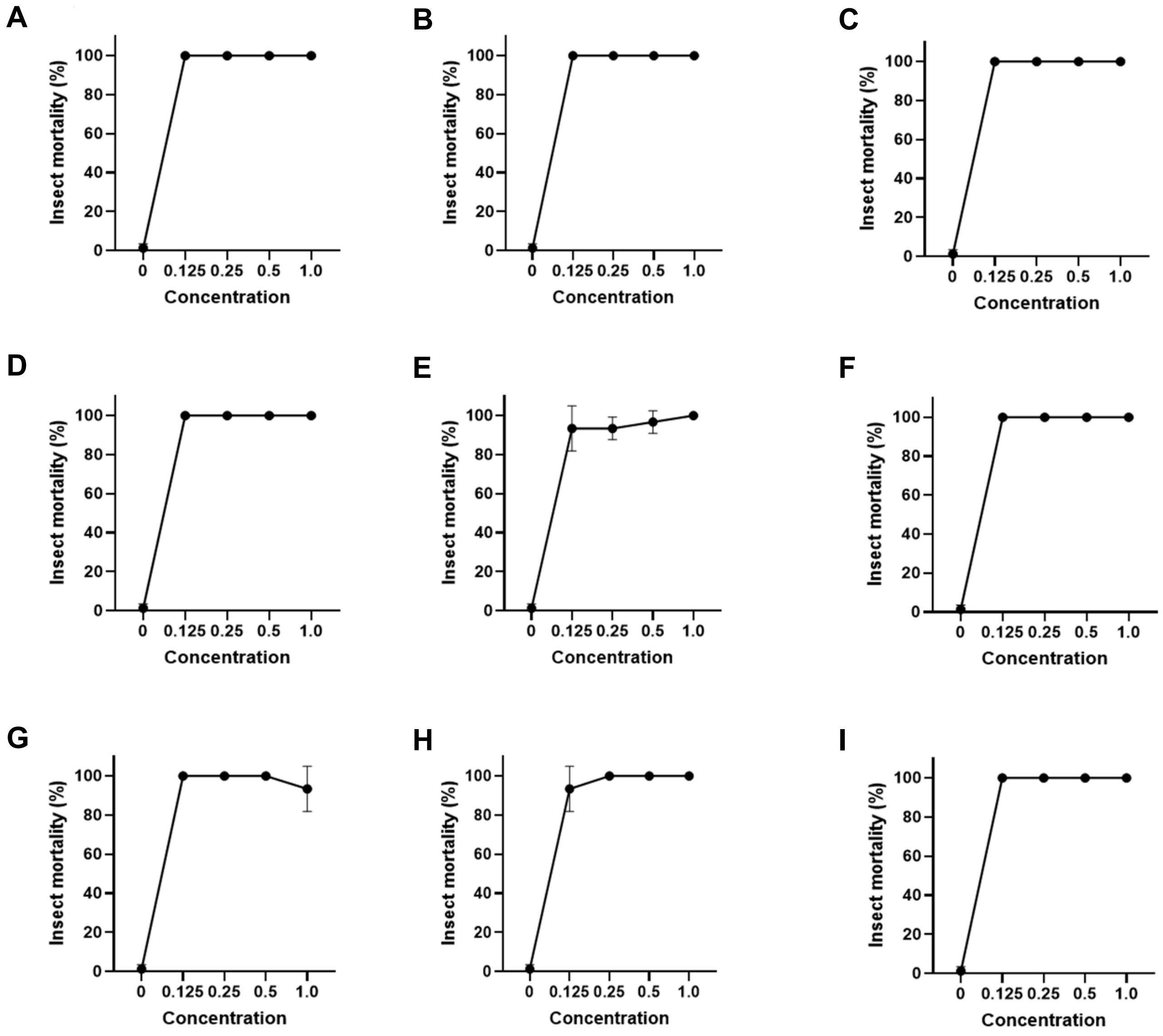

Figure S8. Mortality rate (%) of waxworm larvae 48h after injecting different *Xenorhabdus* strains at various cell concentrations (OD_{600} = 0.125, 0.25, 0.5 and 1.0). A) *X. bharatensis* sp. nov. CS20^T B) *X. entomophaga* sp. nov. XENO-11^T; C) *X. griffiniae* DSM 17911^T; D) *X. indica* DSM 17382^T; E) *X. innexi* DSM 16336^T; F) *X. khoisanae* DSM 25463^T; G) *X. siamensis* sp. nov. AUT15.5^T; H) *X. stockiae* DSM 17904^T; I) *X. thailandensis* sp. nov. CCN3.3^T.

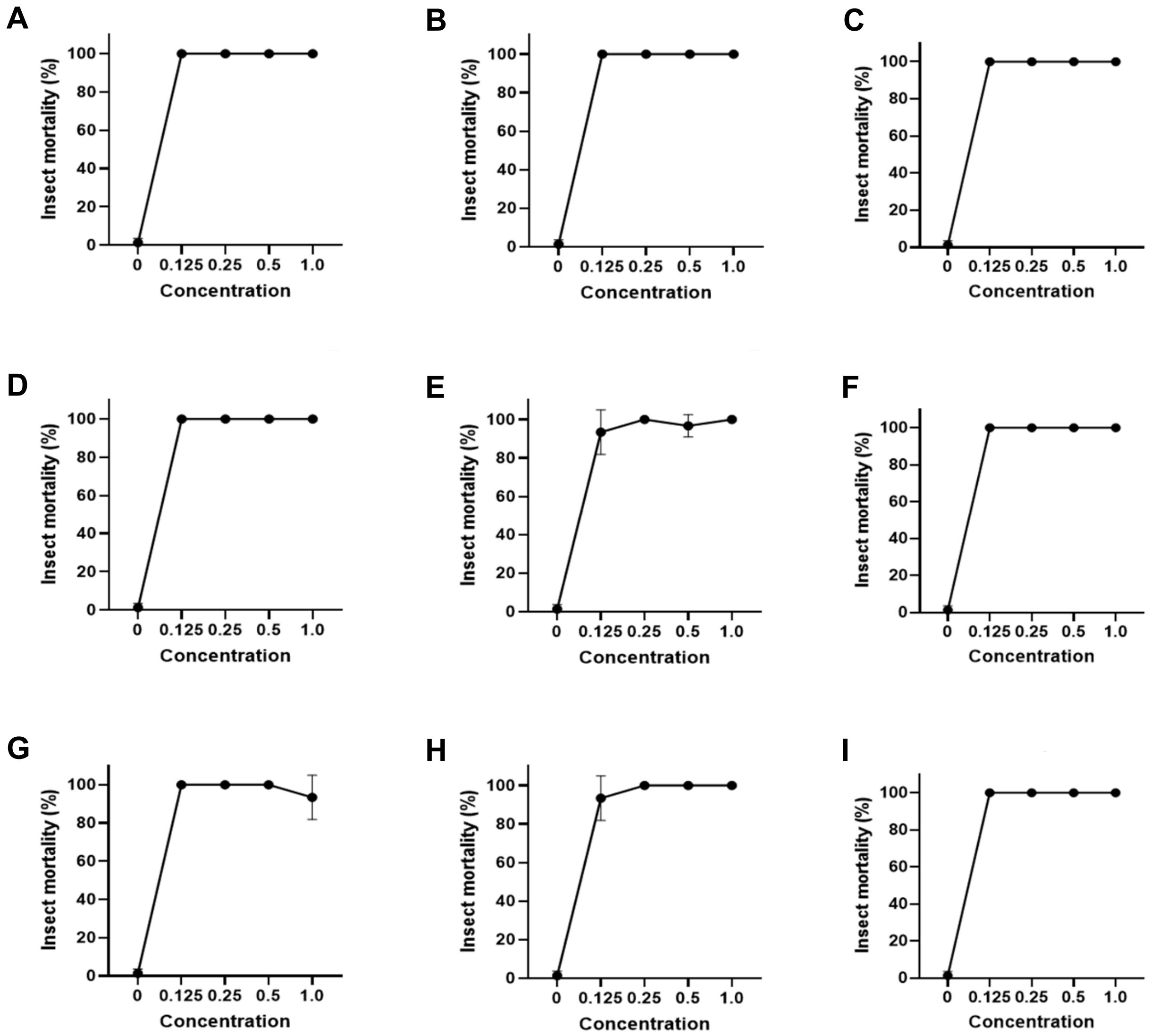

Figure S9. Mortality rate (%) of waxworm larvae 72h after injecting different *Xenorhabdus* strains at various cell concentrations (OD_{600} = 0.125, 0.25, 0.5 and 1.0). A) *X. bharatensis* sp. nov. CS20^T B) *X. entomophaga* sp. nov. XENO-11^T; C) *X. griffiniae* DSM 17911^T; D) *X. indica* DSM 17382^T; E) *X. innexi* DSM 16336^T; F) *X. khoisanae* DSM 25463^T; G) *X. siamensis* sp. nov. AUT15.5^T; H) *X. stockiae* DSM 17904^T; I) *X. thailandensis* sp. nov. CCN3.3^T.

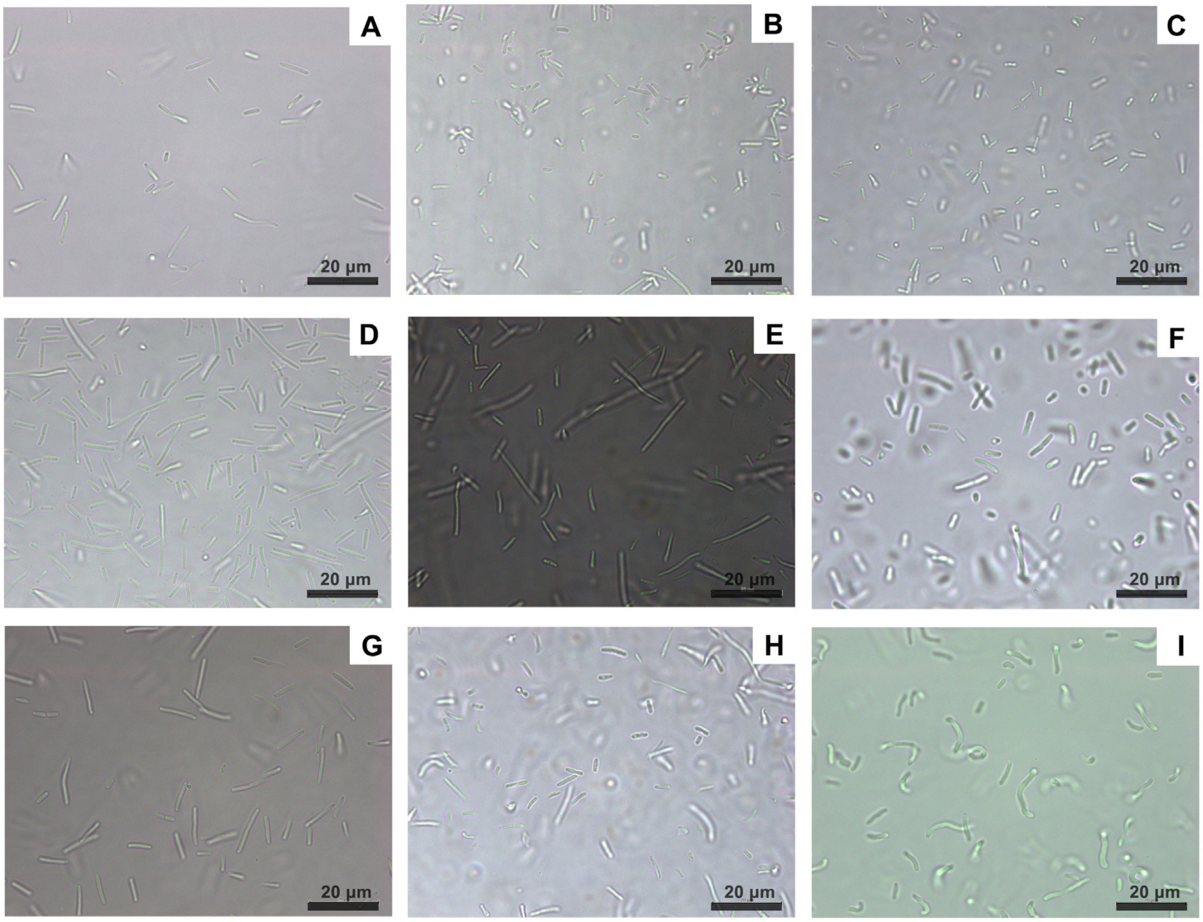

Figure S10. Light microscopy (LM) photographs of the newly described *Xenorhabdus* species and their more closely related species. A) *X. bharatensis* sp. nov. CS20^T B) *X. entomophaga* sp. nov. XENO-11^T; C) *X. griffiniae* DSM 17911^T; D) *X. indica* DSM 17382^T; E) *X. innexi* DSM 16336^T; F) *X. khoisanae* DSM 25463^T; G) *X. siamensis* sp. nov. AUT15.5^T; H) *X. stockiae* DSM 17904^T; I) *X. thailandensis* sp. nov. CCN3.3^T. Bars correspond to 20 µm.

-SUPPLEMENTARY TABLES-

Table S1. National Center for Biotechnology Information (NCBI) accession numbers of the bacterial sequences used in this study. Sequences generated in this study are shown in bold.

Strain	16S	Genome
X. aichiensis XENO-7^T	OQ439939	JAQRFO01
X. anantnagensis XENO-2^T	OQ439938	JAQRFN01
X. bakwenae SF857^T	OQ418033	CP119194.1
X. beddingii Q58^T	AY278675	MUBK01
***X. bharatensis* sp. nov. CS20^T**	**PP544787**	**JBBMXG01**
X. bovienii subsp. *africana* XENO-1^T	ON497255	JAMGSK01
X. bovienii subsp. *bovienii* T228^T	AY278673	JANAIF01
X. budapestensis DSM 16342^T	AJ810293	NIBS01
X. cabanillasii USTX62^T	AY521244	QTUB01
X. doucetiae FRM16^T	DQ211709	FO704550
X. eapokensis DL20^T	KX602187	MKGQ01
X. ehlersii DSM 16337^T	AJ810294	NIBT01
***X. entomophaga* sp. nov. XENO-11^T**	**PP544789**	**JBBMXH01**
X. griffiniae DSM 17911^T	DQ211710	JAQRFM01
X. hominickii KE01^T	DQ211719	NJAI01
X. indica DSM 17382^T	AM040494	NKHP01
X. innexi DSM 16336^T	AJ810292	NIBU01
X. ishibashii GDh7^T	GQ149086	NJAK01
X. japonica DSM 16522^T	D78008	FOVO01
X. khoisanae DSM 25463^T	HQ142625	JAQRFL01
X. koppenhoeferi USNJ01^T	DQ205450	FPBJ01
X. kozodoii SaV^T	DQ211716	NJCX01
X. lircayensis VLS^T	MT466535	JACOII01
X. magdalenensis IMI 397775^T	HQ877464	JAQRFK01
X. mauleonii VC01^T	DQ211715	NITY01
X. miraniensis Q1^T	DQ211713	NITZ01
X. nematophila ATCC 19061^T	AY278674	FN667742
X. poinarii G6^T	D78010	FO704551
X. romanii PR06-A^T	DQ211717	JAQRFJ01
***X. siamensis* sp. nov. AUT15.5^T**	**PP544788**	**JBBMXE01**
X. stockiae DSM 17904^T	DQ202309	NJAJ01
X. szentirmaii DSM 16338^T	AJ810295	NIBV01
***X. thailandensis* sp. nov. CCN3.3^T**	**PP544790**	**JBBMXF01**
X. thuongxuanensis 30TX1^T	KX602193	MKGR01
X. vietnamensis VN01^T	DQ205447	MUBJ01
X. yunnanensis XENO-10^T	OQ439939	JAQRFI01

Table S2. Features of the *Xenorhabdus* genomes used in this study.

Species	Basepairs	Percent G+C	No. proteins
X. aichiensis XENO-7^T	4 699 893	44.63	4196
X. anantnagensis XENO-2^T	4 318 764	42.88	3905
X. bakwenae SF857^T	4 030 269	44.78	3830
X. beddingii Q58^T	4 096 354	45.19	3633
***X. bharatensis* sp. nov. CS20^T**	**4 776 084**	**43.45**	**4228**
X. bovienii subsp. *africana* XENO-1^T	4 674 198	44.73	4217
X. bovienii subsp. *bovienii* T228^T	4 567 727	44.73	4395
X. budapestensis DSM 16342^T	4 311 148	43.13	3686
X. cabanillasii USTX62^T	4 335 622	42.90	3812
X. doucetiae FRM16^T	4 195 202	45.71	3645
X. eapokensis DL20^T	4 242 650	43.49	3757
X. ehlersii DSM 16337^T	4 058 264	43.78	3807
***X. entomophaga* sp. nov. XENO-11^T**	**4 963 997**	**43.88**	**4815**
X. griffiniae DSM 17911^T	4 557 634	43.67	4187
X. hominickii KE01^T	5 335 857	43.39	4871
X. indica DSM 17382^T	4 501 483	42.86	3800
X. innexi DSM 16336^T	4 573 808	43.67	4019
X. ishibashii GDh7^T	3 859 346	42.94	3554
X. japonica DSM 16522^T	3 560 854	42.70	3214
X. khoisanae DSM 25463^T	4 805 416	43.81	4322
X. koppenhoeferi USNJ01^T	3 181 650	43.06	2763
X. kozodoii SaV^T	4 127 775	44.70	3726
X. lircayensis VLS^T	4 279 293	44.15	3626
X. magdalenensis IM139775^T	3 710 083	45.59	3407
X. mauleonii VC01^T	5 117 864	43.90	4428
X. miraniensis Q1^T	4 992 355	43.67	4252
X. nematophila ATCC 19061^T	4 587 837	44.21	4549
X. poinarii G6^T	3 659 523	44.55	3371
X. romanii PR6a^T	3 879 794	45.05	3631
***X. siamensis* sp. nov. AUT15.5^T**	**4 425 965**	**42.77**	**3912**
X. stockiae DSM 17904^T	4 667 712	43.52	4021
X. szentirmaii DSM 16338^T	4 824 775	43.98	4566
***X. thailandensis* sp. nov. CCN3.3^T**	**3 794 703**	**43.23**	**3508**
X. thuongxuanensis 30TX1^T	3 935 399	43.05	3474
X. vietnamensis VN01^T	4 663 685	42.99	3909
X. yunnanensis XENO-10^T	4 667 994	43.14	4534

Table S3. Features of the genomes of *X. bharatensis* sp. nov. CS20^T, *X. entomophaga* sp. nov. XENO-11^T, *X. siamensis* sp. nov. AUT15.5^T, and *X. thailandensis* sp. nov. CCN3.3^T generated in this study.

	**CS20^T**	**XENO-11^T**	**AUT15.5^T**	**CCN3.3^T**
Scaffold L50	9.00	42	7	3
Scaffold N50	199653	34174	272423	304672
Scaffold L90	25	146	16	11
Scaffold N90	49219	7008	118374	92447
Scaffold len_max	409348	111868	483853	1146418
Scaffold len_min	208	201	18239	3155
Scaffold len_mean	35910	8342	201180	180700
Scaffold len_median	520	704	184451	92447
Scaffold len_std	79715	16925	120236	265911
Scaffold num_A	1350859	1388244	1271733	1077099
Scaffold num_T	1349817	1397561	1261433	1077322
Scaffold num_C	1045411	1082467	961748	812529
Scaffold num_G	1029997	1095725	931051	827753
Scaffold num_N	0	0	0	0
Scaffold num_bp	4776084	4963997	4425965	3794703
Scaffold num_bp_not_N	4776084	4963997	4425965	3794703
Scaffold num_seq	133	595	22	21
Scaffold GC content overall	43.45	43.88	42.77	43.23

Table S4. Completeness (%) and contamination (%) of the bacterial genomes generated in this study assessed by checkM.

Strain	Completeness (%)	Contamination (%)
X. bharatensis sp. nov. CS20^T	100	0.54
X. entomophaga sp. nov. XENO-11^T	100	0.0
X. siamensis sp. nov. AUT15.5^T	100	1.08
X. thailandensis sp. nov. CCN3.3^T	100	0.0

Table S5. Antibiotic-resistance conferring genes of *X. bharatensis* sp. nov. CS20^T, *X. entomophaga* sp. nov. XENO-11^T, *X. siamensis* sp. nov. AUT15.5^T, and *X. thailandensis* sp. nov. CCN3.3^T. (+): present; (-): absent or non-functional.

Gene	Resistance Mechanism	AMR Gene Family	Drug Class	*X. bharatensis* sp. nov. CS20^T	*X. entomophaga* sp. nov. XENO-11^T	*X. siamensis* sp. nov. AUT15.5^T	*X. thailandensis* sp. nov. CCN3.3^T
KpnH	Antibiotic efflux	Major facilitator superfamily (MFS) antibiotic efflux pump	Macrolide antibiotic, fluoroquinolone antibiotic, aminoglycoside antibiotic, carbapenem, cephalosporin, penam, peptide antibiotic	+	+	+	+
rsmA		Resistance-Nodulation-Cell Division (RND) Antibiotic Efflux Pump	Fluoroquinolone antibiotic, diaminopyrimidine antibiotic, phenicol antibiotic	+	+	+	+
CRP			Macrolide antibiotic, fluoroquinolone antibiotic, penam	+	+	+	+
adeF			fluoroquinolone antibiotic, tetracycline antibiotic	+	-	-	-
KpnF		Small Multidrug Resistance (SMR) Antibiotic Efflux Pump	Macrolide antibiotic, aminoglycoside antibiotic, cephalosporin, tetracycline antibiotic, peptide antibiotic, rifamycin antibiotic, disinfecting agents and antiseptics	-	+	+	+
qacJ			Disinfecting agents and antiseptics	-	-	+	+
vanT	Antibiotic target alteration	Glycopeptide Resistance Gene Cluster	Glycopeptide antibiotic	+	+	+	+
EF-Tu		Elfamycin resistant EF-Tu	Elfamycin antibiotic	-	+	+	+
PBP3		Penicillin-Binding Protein Mutations Conferring Resistance to Beta-Lactam Antibiotics	Cephalosporin, cephamycin, penam	-	-	+	+
ArnT		Phosphoethanolamine Transferase	Peptide antibiotic	+	+	+	+
FosG	Antibiotic inactivation	Fosfomycin Thiol Transferase	Phosphonic acid antibiotic	-	-	-	+
FosA8		Fosfomycin Thiol Transferase		-	-	-	+
